# GDF11-secreting cell transplant efficiently ameliorates age-related pulmonary fibrosis

**DOI:** 10.1016/j.ymthe.2025.07.003

**Published:** 2025-07-16

**Authors:** Li Guo, Pascal Duchesneau, Eric D. Jong, Evan Sawula, Chengjin Li, Thomas K. Waddell, Andras Nagy

**Affiliations:** 1 Lunenfeld-Tanenbaum Research Institute, Sinai Health System, Toronto, ON M5T 3H7, Canada; 2 Division of Thoracic Surgery, Toronto General Hospital Research Institute, University Health Network, University of Toronto, Toronto, ON M5G 2C4, Canada; 3 Institute of Medical Science, University of Toronto, Toronto, ON M5S 3H2, Canada; 4 Department of Obstetrics & Gynecology, Faculty of Medicine, University of Toronto, Toronto, ON M5G 1E2, Canada

**Keywords:** cell and gene therapy, GDF11, inducible expression, senescence attenuation, pluripotent stem cells, SafeCell genome editing, lung progenitors, alveolar regeneration, idiopathic pulmonary fibrosis, fibrosis resolution

## Abstract

Here, we present a combination of cell and gene therapy that harnesses the regenerative properties of GDF11 in age-related pulmonary fibrosis. Our genome-edited SafeCell-GDF11 mouse embryonic stem cell line provides controlled proliferation and efficient derivation to lung progenitors while inducibly expressing GDF11. When these cells were transplanted into bleomycin-injured aged mice, they acted as a source of reparative cells, restoring the damaged alveolar epithelium. Furthermore, the transplanted cells acted as an “*in situ* factory,” enabling the production of GDF11 in response to the inducer drug. This approach attenuated age-associated senescence and led to the successful resolution of fibrosis. Our study presents a GDF11-expressing cell-based strategy that demonstrates the feasibility of promoting alveolar regeneration in a mouse model of age-related pulmonary fibrosis. Additionally, this approach offers a versatile tool that can be expanded to incorporate other regenerative and anti-aging factors. This helps overcome limitations such as high production costs and a short half-life of therapeutic factors. One of the strengths of our system is its ability to allow precise regulation of factor expression when needed to address specific aging phenotypes.

## Graphical abstract

**Figure:**
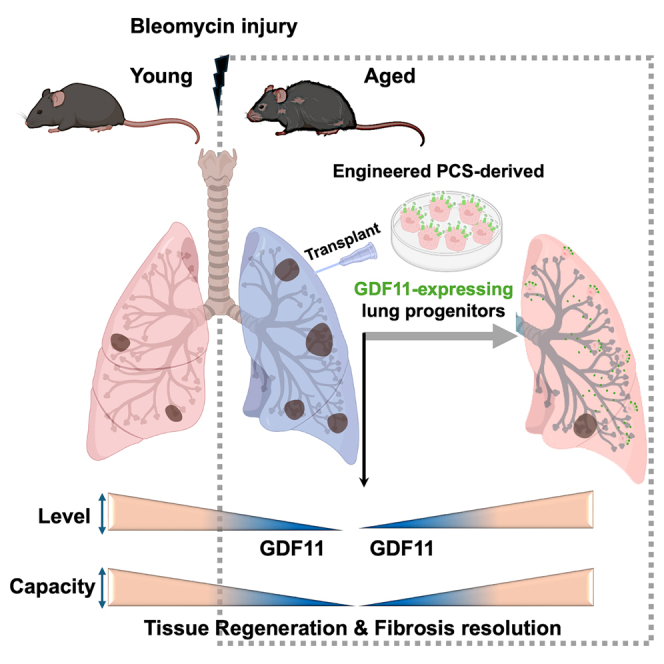

## Teaser

Nagy and colleagues present a combined cell and gene therapy approach to target age-related pulmonary fibrosis using engineered lung progenitors with inducible expression of GDF11. These cells repair alveoli, serve as an *in situ* GDF11 secretion source, and reduce fibrosis, offering a platform for controlled, localized delivery of regenerative factors.

## Introduction

As we age, our tissues undergo degenerative changes that are closely linked to the development of chronic diseases and cancer. This degeneration becomes increasingly pronounced as our body’s regenerative capabilities diminish, leading to a decline in organ function and overall health. To address this, it is vital to bolster natural regeneration processes that can facilitate injury recovery and slow the advancement of age-related disease conditions.

Discovering or engineering factors that promote regeneration and suppress degeneration could lead to effective medical treatments for restoring health in situations where natural healing is insufficient. A multitude of factors, including sirtuins (SIRT),^1^^,^^2^ Klotho,^3^^,^^4^^,^^5^ insulin-like growth factor 1 (IGF-1),^6^^,^^7^^,^^8^ platelet factor 4 (PF4),^9^ and growth differentiation factor 11 (GDF11),^10^^,^^11^^,^^12^ have been proposed as prospective regenerative or anti-aging agents. GDF11, in particular, has been extensively researched for its role in upholding organ function and promoting regeneration in various diseases.^13^ However, investigations into GDF11’s efficacy in addressing age-related conditions such as cardiovascular disease, skeletal muscle degeneration, and osteogenesis initially yielded conflicting outcomes.^10^^,^^14^^,^^15^^,^^16^ Indeed, some research suggests that GDF11 possesses anti-fibrotic properties by inhibiting transforming growth factor β1 (TGF-β1) signaling and promoting bone morphogenetic protein (BMP) signaling,^17^^,^^18^^,^^19^ which reduces collagen deposition and fibroblast activation regarding fibrosis. Conversely, other studies indicate that it promotes fibrosis by enhancing fibroblast proliferation and collagen synthesis through the activation of ALK5/Smad3.^16^^,^^20^^,^^21^ The variability in findings is attributed to differences in experimental models, measurement challenges due to structural similarities with GDF8,^22^^,^^23^ and dose-dependent effects in which low levels may aid repair, while high levels exacerbate fibrotic processes and negatively impact tissue health.^24^ Furthermore, the role of GDF11 may vary at different stages of fibrosis.^18^^,^^21^ Overall, the complexity of GDF11’s signaling and context-dependent actions highlights the necessity for further research such as ours to clarify its therapeutic potential and resolve existing discrepancies.

By now, the controversy surrounding GDF11 has been mostly resolved by discovering tissue-specific variation in its expression and function.^13^ Recent reviews have highlighted that the dispute around GDF11 function is presumably rooted in its diverse effects, which depend on the tissue, organ system, and disease-specific disparities in GDF11 expression.^25^^,^^26^ A further positive development is that there has been a significant increase in publications demonstrating the therapeutic effects of GDF11 in treating various conditions.^12^^,^^27^^,^^28^^,^^29^ The current understanding is that GDF11 is unlikely to be the sole mediator of regeneration, but it does play a role in specific conditions and organ functions.

Idiopathic pulmonary fibrosis (IPF) is a devastating lung disease with limited treatment options. The age-related decline in lung function significantly contributes to the development of IPF,^30^^,^^31^^,^^32^ reducing therepair capabilities and resolving fibrosis. This leads to irreparable tissue damage, further impaired function, and high mortality rates. Therefore, it is crucial to develop strategies to counteract age-related decline in lung function and promote effective restoration of homeostasis.

The bleomycin (BLM)-induced lung fibrosis model in mice is widely used for studying IPF. It includes an initial phase of acute inflammation and alveolar injury, followed by progressive fibrosis peaking 14 to 21 days post-BLM administration.^33^^,^^34^ Microcomputed tomography (micro-CT) and histological analyses show that fibrosis resolution aligns with reduced collagen deposition, decreased inflammatory cell infiltration, and normalization of lung architecture.^35^^,^^36^ The fibrotic response is self-limiting, with spontaneous resolution after about 3 weeks, unlike human IPF, which is progressive and irreversible.^33^^,^^37^ This model is valuable for testing antifibrotic therapies, as exemplified by the successful development of nintedanib, which is an effective therapeutic drug for specific stages of fibrosis progression.^34^^,^^37^

The “hallmarks of aging” describe the mechanisms that cause a decrease in regenerative capacity. These hallmarks include stem cell exhaustion, telomere attrition, mitochondrial dysfunction, epigenetic alterations, genomic instability, cellular senescence/apoptosis, defective proteostasis, dysregulated nutrient sensing, and distorted intercellular communication.^38^^,^^39^^,^^40^ Cellular senescence and telomere attrition^41^^,^^42^^,^^43^ are two characteristic signs of aging that are especially relevant to humans with IPF. They present promising targets for regenerative and anti-aging interventions. It remains to be determined whether GDF11 has regenerative and anti-aging effects on the lungs and age-related pulmonary conditions; therefore, we have selected GDF11^44^^,^^45^ as a prototype for our study targeting IPF.

Like other protein-based regenerative molecules, the practical usage of *in vitro*-manufactured recombinant GDF11 (rGDF11) is hampered by its high cost and short half-life (12 h).^46^ Moreover, like many other protein hormones, GDF11’s beneficial effects are only seen in a narrow concentration window. Excessive levels of GDF11 could potentially lead to adverse consequences, such as neurotoxicity, cachexia, and mortality.^24^^,^^47^^,^^48^ These limitations highlight the need to explore alternative approaches that could lead to safe and feasible clinical applications.

An alternative method to circumvent these limitations involves the targeted delivery of therapeutic cells to diseased sites, where they are engineered to secrete GDF11 in response to small-molecule inducers. This approach could enable precise targeting of specific organs or tissue sites. The biological activity of GDF11 is dependent on various factors such as disease pathologies, organ function, affected tissue, and the aging process.^13^^,^^19^^,^^25^ Therefore, a cell transplant-based system that allows for controlled expression of GDF11 is feasible and necessary to achieve therapeutic benefits while minimizing systemic adverse effects. It also permits the dose and duration optimization of the treatment.

We have established embryonic stem cells (ESCs) expressing GDF11 in a doxycycline (dox)-inducible manner. The cells also contained our SafeCell (SC) system^49^ that ensures safety by eliminating harmful, highly proliferative cells through ganciclovir (GCV) treatment prior to or post-transplanting lung progenitors differentiated *in vitro* from these genome-edited ESCs.

We demonstrated that these cells could replenish the pool of alveolar epithelial cells to resolve the disrupted alveolar epithelium and functionally contribute to tissue regeneration. More important, the engrafted cells are an *in situ* “factory” of GDF11, efficiently attenuating age-associated senescence for successful fibrosis resolution.

Our study provides an alternative therapeutic approach to treating age-related pulmonary fibrosis by combining cell and gene therapy. The approach also serves as a therapeutic model for other degenerative diseases, where the use of *in vitro*-produced clinical-grade biologics is limited by high cost, short half-life, and frequent treatment needs.

## Results

### GDF11 expression declines in aging lungs under both physiological health and pathological conditions

In both adult humans and mice, the expression of GDF11 varies across different tissues at both mRNA and protein levels.^13^ Therefore, we first evaluated GDF11 expression in the lung during aging under physiological health and pathological conditions.

**Figure 1:**
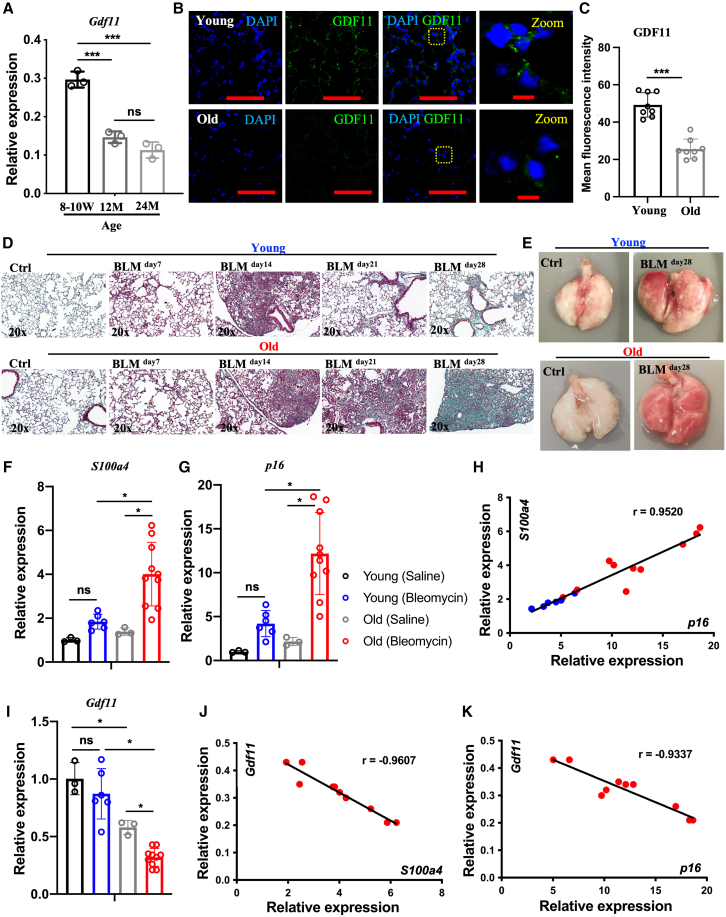
GDF11 expression declines in aging lungs under both physiologically healthy and pathological conditions (A) The expression of *Gdf11* in the lungs from the mice of different ages, as measured by RT-qPCR. (B) Confocal microscopy images of distal airways of young mice (8–10 weeks old) and old mice (12 months old) showing nuclear stain DAPI (blue) and GDF11 (green). (C) Quantification of mean fluorescence intensity of GDF11. (D) Representative images of Masson’s trichrome staining of the lungs of young and old mice after 7, 14, 21, and 28 days of BLM-induced pulmonary fibrosis. Interstitial collagen (blue) persisted in the lungs of old mice 28 days after BLM treatment. (E) Representative images of whole lungs from all the experimental groups at day 28 after BLM treatment. (F and G) The expression of the fibrosis gene *S100a4* (F) and the senescence marker *p16* (G) in the lungs of young and old mice 28 days after saline or BLM treatment, as measured by RT-qPCR comparing fold differences in gene expression in young, healthy controls. (H) The correlation of the expression of *S100a4* with *p16*. (I) The expression of GDF11 in the lungs of young and old mice 28 days after saline or BLM treatment, as measured by RT-qPCR comparing fold differences in gene expression in young, healthy controls. (J and K) The correlation of the expression of *Gdf11* with *S100a4* (J) and *p16* (K) in the group of old mice. ∗*p* < 0.05; ∗∗*p* < 0.001; ∗∗∗*p* < 0.0001. Scale bars, 100 μm (B); 10 μm (B Zoom).

In one group of young (8- to 10-week-old) and two groups of old mice (12- and 24-month-old), *Gdf11* lung expression levels were compared using RT-qPCR (Figure S1). While the expression level of the young mice was significantly higher than that of the old mice, the two old groups did not differ significantly. Therefore, the 12-month age was chosen for the “old” group (Figure 1A) in the studies presented. The RT-qPCR analysis-based findings were supported by immunohistochemistry, showing the decreased level of GDF11 protein in the distal lung (Figures 1B and 1C).

BLM-induced injury predominantly targets the distal lung, leading to basement membrane disruption and fibrosis development in the alveolar compartment.^50^ While not a perfect model of human IPF, this model mimics age-related deteriorations in lung function, including enhanced p16 activation indicative of senescence.^51^ Our study corroborated earlier findings^52^ by demonstrating that old mice exhibited impaired fibrosis resolution compared to the nearly complete recovery seen in young mice on day 28 post-BLM injury (Figures 1D–1F). Thus, given the lingering fibrosis in old mice, we selected day 28 to evaluate lung GDF11 expression.

Age-dependent accumulation of senescence has been implicated in human IPF.^51^ Our study revealed sustained elevation of the senescence marker *p16* in old mice at day 28 post-BLM administration compared to young mice (Figure 1G). Additionally, we observed a strong correlation (*r* = 0.9520) between *p16* levels and the fibrosis marker *S100a4* expression (Figure 1H), indicating a close relationship between senescence and fibrosis severity.

Compared to healthy controls, BLM-injured old mice showed decreased *Gdf11* levels, suggesting an accelerated age-related decline of *Gdf11* due to BLM injury (Figure 1I). Additionally, we found significant negative correlations between *Gdf11* levels and *S100a4* expression (*r* = −0.9607) (Figure 1J) and *p16* (*r* = −0.9337) (Figure 1K). These findings indicate that GDF11 levels decrease in aging lungs under both normal physiological and BLM-induced pathological conditions. This reduction is associated with the impaired fibrosis resolution mediated by cellular senescence.

### Exogenous GDF11 protein administration partially ameliorates age-related deterioration of fibrosis resolution in the distal lung

**Figure 2:**
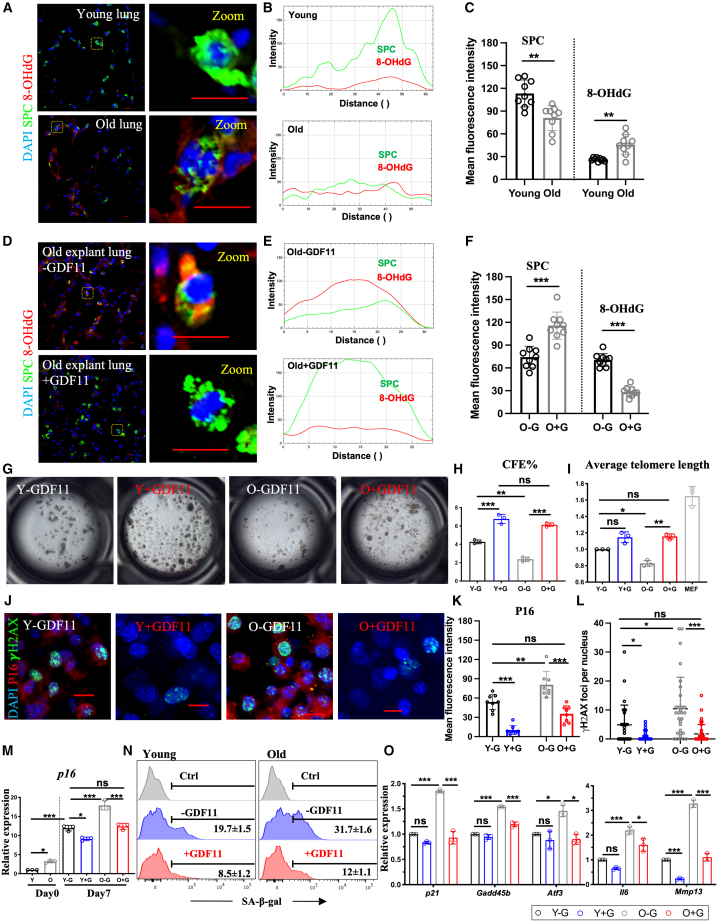
Exogenous GDF11 can partially ameliorate age-related cellular deterioration in the distal lungs (A) Confocal microscopy images of distal airways of young mice (8–10 weeks old) and old mice (12 months old) showing nuclear stain DAPI (blue), SPC (green), and 8-OHdG (red). (B) Fluorescence intensity profile plot of magnified cells. (C) Quantification of fluorescence intensity of SPC and 8-OHdG. (D) Representative confocal microscopy images of distal airways of old explant lungs treated with or without GDF11 protein showing nuclear stain DAPI (blue), SPC (green), and 8-OHdG (red). (E) Fluorescence intensity profile plot of magnified cells. (F) Quantification of fluorescence intensity of SPC and 8-OHdG. (G) Bright-field images depicting the generation of colonies derived from AEC-IIs isolated from young and old mice in the absence and presence of GDF11 recombinant protein. (H) The colony-forming efficiency. (I) Expression levels of average telomere length in cells obtained from each condition, as measured by RT-qPCR comparing fold differences in the expression in control young cells. (J) Representative confocal microscopy images of AEC-IIs isolated from young and old mice in the absence and presence of GDF11 recombinant protein showing nuclear stain DAPI (blue), γH2AX (green), and P16 (red). (K and L) Quantification of fluorescence intensity of P16 (K) and γH2AX foci (L). (M) Expression levels of *p16* in the AEC-IIs obtained from each condition, as measured by RT-qPCR comparing fold differences in the expression in day 0 fresh AEC-II cells of young mice. (N) Flow cytometric analysis of SA-β-gal expression levels in cells cultured in each condition. Data are representative of a minimum of three independent biological replicates. (O) Expression levels of age-related stress-response genes in the p53 tumor suppressor pathway in cells obtained from each condition, as measured by RT-qPCR comparing fold differences in the expression in control young cells. ∗*p* < 0.05; ∗∗*p* < 0.001; ∗∗∗*p* < 0.0001. Scale bars, 10 μm (A, D, and J).

Alveolar type II cells (AEC-IIs) in the distal lung, akin to stem cells in other tissues,^53^^,^^54^ show diminished regenerative capability with age. This decline, exacerbated by protein homeostasis disruptions, results in cellular harm and tissue dysfunction.^55^^,^^56^ Our investigation into aging’s impact on AEC-IIs began with analyzing surfactant protein C (SPC) expression, a crucial protein for AEC-II maintenance. As anticipated, old lungs exhibited reduced SPC protein levels (Figures 2A–2C).

Moreover, we explored age-related mitochondrial DNA (mtDNA) damage, known to impact the reduced lifespan of lung progenitor cells.^57^^,^^58^^,^^59^ The accumulation of mtDNA damage is a hallmark of aging and is linked to various age-related pulmonary diseases, such as IPF, chronic obstructive pulmonary disease, and lung cancer. mtDNA damage induces cellular senescence and apoptosis, contributing to dysfunction in AEC-IIs and the progression of these diseases.^58^^,^^59^ To assess the extent of mtDNA damage in the aging distal lung, we conducted an immunohistochemical analysis using an 8-hydroxy-guanosine (8-OHdG) antibody to label DNA damage in both the mitochondria and nucleus.^60^ The results showed a significant increase in the number and immunoactivity of 8-OHdG^+^ cells in the old lungs, indicating that mtDNA damage accumulates with aging in the distal lung, including the AEC-II compartment (Figures 2A–2C).

We found that treatment with recombinant GDF11 halted the age-related decrease in SPC expression (Figures 2D–2F), with no detectable abnormalities (Figure S2C). Regarding the mtDNA damage, we observed that in the explanted cultured lungs, 8-OHdG expression increased compared to fresh lungs, indicating a worsening of mtDNA damage due to the oxidative stress of the culture environment. Then, we treated the old lung explants with 50 ng/mL^61^ GDF11 recombinant protein daily for up to 7 days (Figure S2A). Although treatment with GDF11 for 3 days did not show significant effects (Figure S2B), remarkably, after 7 days of GDF11 treatment, immunoactivity of 8-OHdG was greatly reduced in the distal lung and SPC+AEC-II cells, suggesting GDF11’s potential in alleviating mtDNA damage from aging- and culture-induced oxidative stress.

As many hallmarks of aging in chronic lung diseases are linked to AEC-II cells, reversing their aging traits could potentially retain the regenerative capability of the young in aging lungs. Therefore, we evaluated the self-renewal potential of AEC-II cells obtained from young (8- to 10-week-old) and old (12-month-old) mice using a colony-forming assay.^62^ AEC-II cells from old mice showed decreased clonogenicity, but treatment with GDF11 protein significantly improved colony-forming efficiency in both age groups (Figures 2G and 2H).

Given that telomere shortening contributes to age-related decline in self-renewal and cellular senescence,^63^^,^^64^ we investigated whether the telomeres were shortened in old AEC-II cells. Indeed, our analysis showed that both the distal lung tissues (Figure S2D) and AEC-IIs (Figure 2I) from old mice had shorter telomeres than those from young mice. Treatment with GDF11 led to significant telomere elongation in aged AEC-II cells. In contrast, there was no significant change in telomere length in young AEC-II cells treated with GDF11, suggesting that the improved clonogenicity of the young may result from reduced *in vitro* oxidative stress (Figure 2I).

Genomic integrity is a crucial factor in cellular health, especially in the presence of aging-related genetic abnormalities.^39^ Repairing DNA double-strand breaks during DNA replication or caused by environmental factors is a significant challenge. Our immunofluorescence analysis of phosphorylated histone H2AX (γH2AX) in lung cells, as a marker of double-strand breaks that increase with aging,^39^ revealed a significant rise in both the number and intensity of positive foci in aged lung cells. Interestingly, the treatment with GDF11 reduced the level of γH2AX immunoreactivity in these cells compared to untreated cells (Figures 2J and 2L).

Furthermore, GDF11 successfully reduced p16 expression and the senescence marker senescence-associated β-galactosidase (SA-β-gal) activity during *in vitro* culture of lung tissue (Figures 2J, 2K, 2M, and 2N). Treatment with GDF11 had a similar effect on stress response genes such as *p21*, *Gadd45b*, *Atf3*, *Interleukin-6* (*Il6*), and the metalloprotease *Mmp13* in the p53 tumor suppressor pathway. We detected an increase in the expression of these genes in the old lung that was partially reversed by GDF11 treatment (Figure 2O).

Clearing senescent cells through apoptosis induction has been shown to aid in resolving fibrosis.^43^ Therefore, we investigated whether GDF11’s ability to counteract senescence in lung cells is linked to inducing apoptosis in senescent cells or inhibiting cellular senescence. Flow cytometric analysis revealed a higher percentage of annexin^+^ PI^−^ cells in cultured old lung cells (36.0% ± 3.4%) than in young cells (9.2% ± 0.3%), indicating increased susceptibility to apoptosis with age, likely due to oxidative stress from culture conditions. Upon treatment with GDF11, both young and old cells showed a decrease in apoptosis (young group: 5.5% ± 0.8% and old group: -18.6% ± 1.2%) (Figure S2E), suggesting that GDF11 inhibits senescence instead of inducing apoptosis of the senesced cells.

In summary, our initial study using GDF11 recombinant protein showed that GDF11 mitigated some of the age-related deteriorations in the lung.

### Generation of lung progenitors with regulatable expression of GDF11

**Figure 3:**
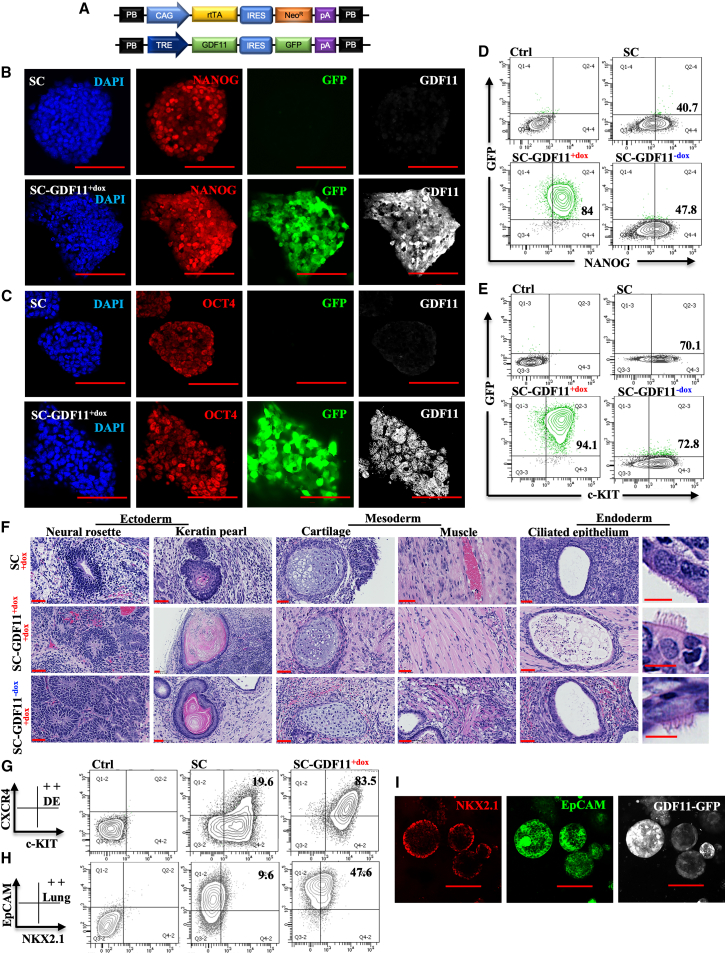
Generation of lung progenitor cells with regulatable production of GDF11 *in vitro* (A) SafeCell mouse ESCs were genetically engineered with a piggyBac transposon to overexpress GDF11 with an GFP fluorescent reporter. (B and C) Confocal microscopy images of parental SC and SC-GDF11^+dox^ ESCs, showing cells stained for NANOG (B) and OCT4 (C) pluripotency markers, GFP reporter, and GDF11. (D and E) Representative flow cytometry contour plots of NANOG (D) and c-KIT (E) expression levels in parental SC and SC-GDF11 ESCs cultured in the presence or absence of dox. (F) Hematoxylin and eosin staining showed the tissue composition of the teratomas, including ectoderm (neural rosette, keratin pearl), mesoderm (cartilage, muscle) and endoderm (ciliated epithelium). (G and H) Representative flow cytometry contour plots of c-KIT^+^CXCR4^+^ cells representing definitive endoderm (G) and NKX2.1^+^EpCAM^+^ lung progenitors (H) during the differentiation of parental SC and SC-GDF11 mouse ESCs. (I) Confocal microscopy images showing lung progenitor cells derived from SC-GDF11 ESCs stained with the nuclear stain NKX2.1 (red), EpCAM (green), and GFP reporter (gray). In (D), (E), (G), and (H), data are representative of a minimum of three independent biological replicates. Scale bar, 100 μm (B, C, and I); 50 μm and 10 μm in zoom (F).

Our SC ESC line^49^ was engineered with dox-inducible GDF11 transgene expression (Figure 3A) using piggyBac transposon technology.^65^ Fourteen clonal lines were derived from single cells expressing GDF11 in a dox-inducible manner, identified via the GFP reporter linked to transgene expression. We screened and thoroughly evaluated these lines for potential variations in inducible GDF11 expression and their commitment to lung progenitor cell fate. The clone that demonstrated the highest effectiveness was selected for further studies and designated as SC-GDF11 cells. The SC system ensures safety by eliminating unwanted dividing cells pre- and post-transplantation, depending on the timing of the GCV treatment.^49^

We analyzed the pluripotency of SC-GDF11 ESCs with and without dox-induced activation of exogenous GDF11 in both *in vitro* and *in vivo* settings. *In vitro*, all ESC lines expressed pluripotency-related markers, such as NANOG (Figures 3B and 3D), OCT4 (Figure 3C) and c-KIT (Figure 3E). Notably, GDF11 expression is fully reversible and tightly regulated by dox, with no detectable leakiness. Flow cytometric analysis of GDF11-GFP expression demonstrates this precise control—showing activation in the presence of dox (SC-GDF11^**+dox**^) and suppression upon its withdrawal (SC-GDF11^−**dox**^), as depicted on the *y* axis of Figures 3D and 3E. This regulation was further confirmed by western blot analysis (data not shown). *In vivo*, the cells formed teratoma in all mice utilized, displaying all three embryonic germ layers (Figure 3F). More important, these cells efficiently differentiated into CXCR4^+^, c-KIT^+^ definitive endoderm and NKX2.1^+^, epithelial cell adhesion molecule-positive (EpCAM^+^) lung progenitors (Figures 3G–3I).

### Exogenous GDF11 alleviates age-related and BLM-induced aggravated cellular senescence

Hence, we conducted an *in vitro* experiment utilizing BLM-induced accelerated cellular senescence^41^^,^^43^ to study the potential anti-senescence properties of different sources of GDF11 on aging lung cells. Of the sources, there were recombinant proteins (rGDF11) and two cell types, such as mouse embryonic fibroblasts (MEFs), which naturally express a certain level of endogenous GDF11^13^ and the SC-GDF11 cells.

**Figure 4:**
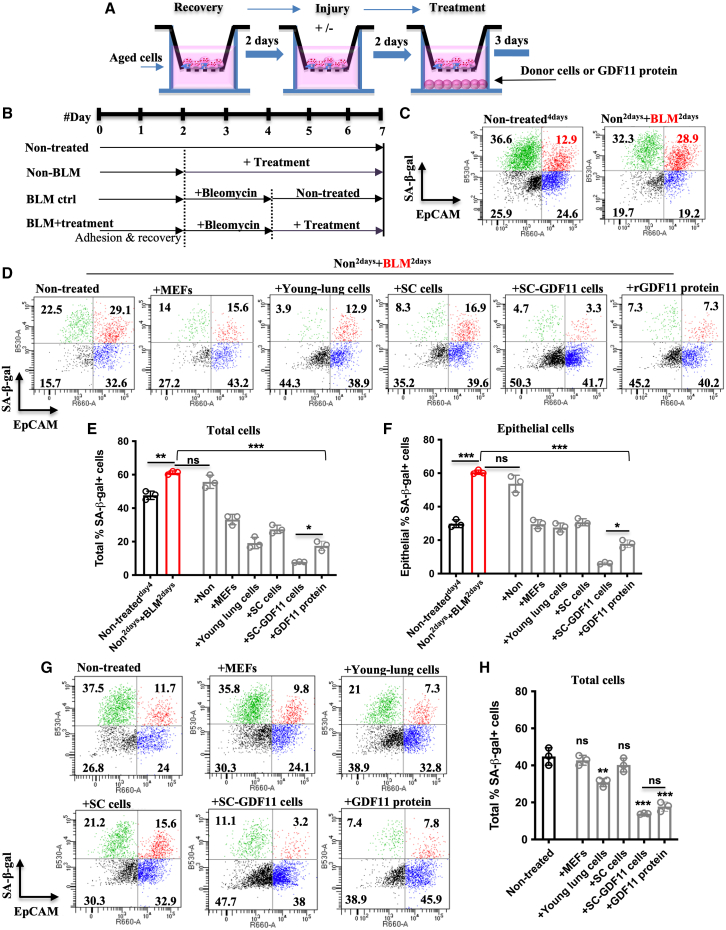
Exogenous GDF11 alleviates age-related and BLM-induced aggravated cellular senescence (A and B) Experimental scheme of the *in vitro* study to evaluate the effect of therapeutic cell-secreted GDF11 on age-related (A) and BLM-induced (B) aggravated cellular senescence. (C) Representative flow cytometry dot plots of SA-β-gal and EpCAM expression levels showing BLM-induced aggravated cellular senescence in distal epithelial cells. (D) The effect of donor cells and rGDF11 on the senescent (SA-β-gal) population. (E and F) Quantification of senescence SA-β-gal^+^ cells in total (E) and epithelial (F) populations obtained from different groups. (G and H) Representative flow cytometry dot plots (G) and quantification (H) showing the effect of donor cells and rGDF11 on the senescence of non-injured cells. ∗*p* < 0.05; ∗∗*p* < 0.001; ∗∗∗*p* < 0.0001. In (C), (D), and (G), data are representative of a minimum of three independent biological replicates.

CD45^-^CD31^-^ cells were freshly isolated from the distal region of the lung, which are the main targets of BLM in alveolar epithelial and fibroblast cells. The cells were then seeded on top of the transwell membrane and allowed to recover for 2 days before subsequent procedures (Figure 4A). To simulate BLM-injury pathological conditions, cells were exposed to a sublethal concentration of BLM (50 μg/mL) on day 3 for 2 days. Then, the cells were maintained in culture for an additional 3 days, either in the presence or absence of rGDF11 or GDF11-producing cells. Concurrently, we examined the response of non-injured cells to these treatments (Figure 4B).

Previous studies found^41^^,^^43^ that BLM-induced severe cellular senescence resulted in a significant increase in SA-β-gal expression in cells, particularly in the alveolar epithelial compartment marked by EpCAM expression (Figures 4C–4F). Compared to controls, treatments with rGDF11 or SC-GDF11^**+dox**^ donor cells after BLM injury demonstrated anti-senescence effects, where the latter even surpassed the rGDF11 effect. However, the treatment with SC cells, MEFs, and young lung cells showed only a moderate reduction in SA-β-gal activity (Figures 4D–4F). No significant changes were observed in SA-β-gal activity in cells treated with SC cells or MEFs compared to the non-treated controls. Treatments with young lung cells were moderately effective compared to the rGDF11 protein and SC-GDF11^**+dox**^ cell-treated groups. The remarkable senescence reversal of these two treatments with GDF11 was equally efficient (Figures 4G and 4H).

### Transplanted lung progenitor cells with the regulatable secretion of GDF11 accelerate fibrosis resolution in aged mice

**Figure 5:**
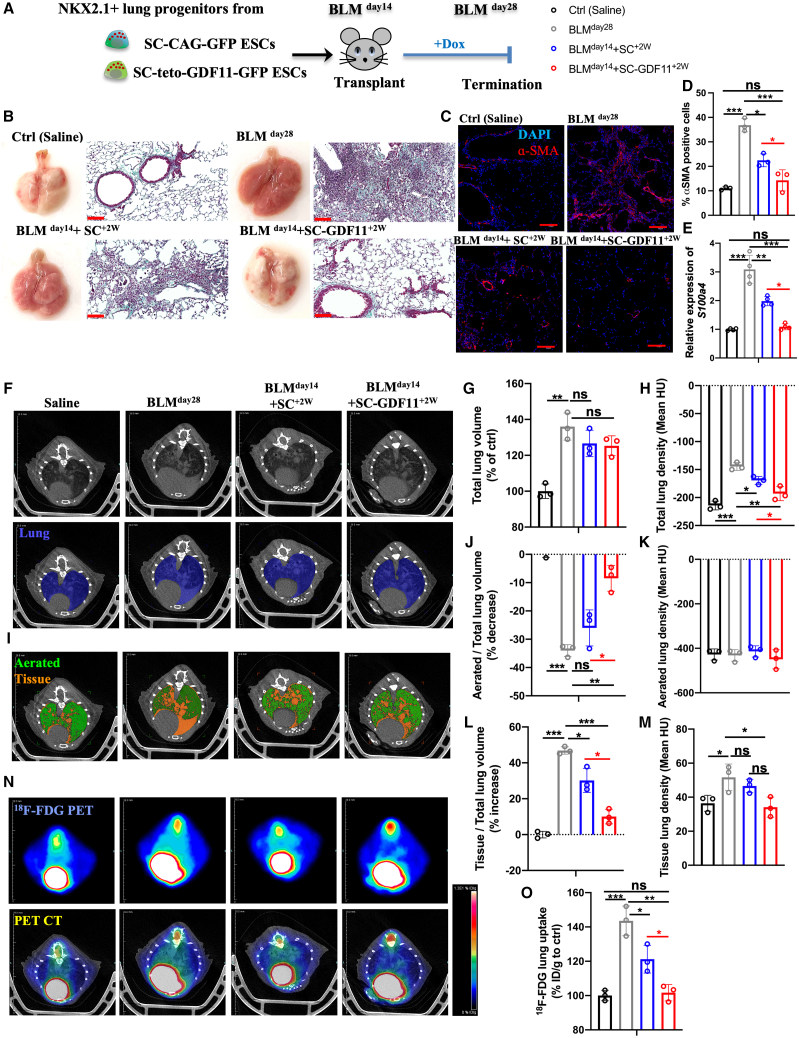
*In vivo* validation of the therapeutic utility of engineered cells for fibrosis (A) Experimental scheme of the *in vivo* study to evaluate the effect of therapeutic cell-secreted GDF11 on fibrosis resolution in the mouse IPF model. (B) Representative images of whole-lung appearance (left) and Masson’s trichrome staining (right) from all the experimental groups after 28 days of BLM-induced pulmonary fibrosis. (C) Representative confocal microscopy images of distal airways of all the experimental lungs showing nuclear stain DAPI (blue) and α-SMA (red). (D) Quantification of α-SMA^+^ cells. (E) The expression of *S100a4* in the lungs of different groups 28 days after saline or BLM treatment, as measured by RT-qPCR comparing fold differences in gene expression in non-injured healthy controls. (F) Representative micro-CT scan images and segmentation of the lung component of different groups. (G and H) Quantifications of the total lung volume (G) and density (H). (I) Segmentation of aerated (green) and tissue (orange) components within the lungs. (J–M) Quantifications of changes in aerated and tissue lung volume (J and L) and lung density (K and M). (N) Representative PET-CT images showing a remarkable improvement in fibrosis resolution in the lungs treated with engineered cells. (O) Quantification of ^18^F-FDG uptake in all experimental groups showing a significant reduction in ^18^F-FDG uptake in the lungs treated with engineered cells. ∗*p* < 0.05; ∗∗*p* < 0.001; ∗∗∗*p* < 0.0001. Scale bar, 100 μm (C).

In this proof-of-principle study, we used the BLM model in aged mice to investigate the effect of transplanted GDF11-expressing, SC-GDF11 cell-derived, NKX2.1^+^ lung progenitors on fibrosis resolution. For controls, NKX2.1^+^ lung progenitors were derived from the SC line, which was marked with constitutively expressed GFP, while in the SC-GDF11 line, the GFP was linked to dox-inducible GDF11 expression (Figure 5A).

The mouse model of BLM-induced lung injury can be divided into three phases. The first is the inflammatory phase, which typically peaks around post-BLM day 7, followed by the fibrotic phase starting around post-BLM day 14 after the first phase subsides. The third phase, fibrosis resolution, shows a significant age-dependent delay in onset and progression.^52^

We investigated whether dox, which has known anti-inflammatory effects, affected fibrosis resolution when administered at the onset of the fibrotic phase (post-BLM day 14). The fibrosis resolution in mice remained unaffected, as assessed at day 28 post-BLM-induced injury (Figure S3), consistent with a previous study.^66^

At the onset of the fibrotic phase (BLM^day14^), we introduced NKX2.1^+^ progenitor cells to injured aged mice transtracheally. Prior to transplantation, the NKX2.1^+^ progenitors were treated with GCV *in vitro* as a safety measure to eliminate highly proliferative potentially tumorigenic cells by the SC system and then purified based on their surface expression of carboxypeptidase M (CPM).^67^

Animals treated solely with saline or BLM, without cell transplantation, served as the negative and positive controls, respectively. The animals were continuously monitored *in vivo* and sacrificed at post-BLM day 28 for further assessments (Figure 5A). The lungs of mice treated with SC-GDF11-ESC-derived cells and kept on a dox diet for 2 weeks thereafter (designated as BLM^day14^+SC-GDF11^+2W^) showed significant improvement in their external appearance compared to that of the SC-ESC-derived donor cell-treated lungs (BLM^day14^+SC^+2W^). The results from *in vivo* imaging validation were consistent with the findings from histological analysis, which revealed that the GDF11-expressing cell-treated lungs had normal alveolar architecture, fewer fibrotic lesions, and less collagen deposition in the parenchyma (Figures 5B and S4A). Immunostaining analysis of myofibroblast marker α-smooth muscle actin (α-SMA) further indicated that the fibrosis was efficiently resolved in GDF11 expression cell-treated lungs (Figures 5C and 5D). Accordingly, the expression of *S100a4*, a marker correlated with fibrosis and its progression, was also significantly reduced (Figure 5E).

Then, micro-CT scans were utilized to conduct qualitative and quantitative assessments of fibrosis. To do this, we segmented the lung (shown as a blue overlay) within the thoracic cavity to measure total lung volume and mean density (Figure 5F). BLM-induced inflammation and progressive fibrosis resulted in an increase in total lung volume compared to saline controls, which remained even after cell treatment (Figure 5G). However, lungs treated with both groups of donor cells showed decreased mean lung density compared to sham-treated BLM lungs. Notably, a significant improvement was observed in the lungs treated with GDF11-expressing cells. The density value was similar to that observed in the saline-control lungs (Figure 5H).

As demonstrated previously, improved lung function does not always accompany normalization of the enlarged total lung volume.^68^ To gain further insights, the lungs were further segmented into aerated (shown as a green overlay) and tissue lung contents (shown as an orange overlay) using thresholding operations, and their respective proportions were quantified for comparison (Figure 5I). The analysis revealed bulk changes in air and tissue contents of the lungs as a whole following the cell treatments. Treatment with GDF11-expressing cells yielded more prominent results, showing a significant increase in aerated lung volume (Figure 5J) and a decrease in tissue lung volume (Figure 5L) and tissue lung density (Figures 5K and 5M), compared to the modest beneficial effect of non-GDF11 transgenic cells in alleviating fibrosis.

Fluorine-18 fluorodeoxyglucose-positron emission tomography-CT (^18^F-FDG PET-CT) imaging has become increasingly popular in recent times for the detection of lung fibrosis and addressing the effectiveness of anti-fibrotic therapies.^69^ The increased uptake of ^18^F-FDG in the lung is due to the upregulation of glucose transporters on myofibroblasts during the fibrotic phase. Our findings were consistent with previous publications, showing a significant increase in ^18^F-FDG uptake in BLM-injured lungs compared to healthy controls. However, after cell treatments, we observed a reduction in ^18^F-FDG uptake in injured lungs, with a greater reduction observed in lungs treated with GDF11-expressing donor cells (Figures 5N and 5O).

### GDF11-expressing donor cells attenuate senescence in aged lungs, achieving fibrosis resolution

Persistent fibrosis is a condition where there is a significant buildup of fibroblasts that are senescent and resistant to apoptosis. Eliminating these senescent fibroblasts can potentially improve lung function and overall physical health in aged mice with BLM-induced lung injury.^43^ Additionally, in both human IPF and BLM-injured aged mice,^41^^,^^42^^,^^43^^,^^70^ age-related senescence in AEC-II cells has been linked to impaired fibrosis resolution in the lungs. Therefore, it is crucial to target and eliminate senescent features in both fibroblasts and AEC-II cells to develop effective therapies against pulmonary fibrosis, potentially halting or reversing its progression in aged populations.

Our *in vitro* study has shown that GDF11-expressing donor cells have a positive impact on age-related and BLM-induced cellular senescence in fibroblasts and AEC-II cells (Figures 4D–4F). Here, we investigated whether these anti-senescence beneficial effects also exist long term in *in vivo* applications.

**Figure 6:**
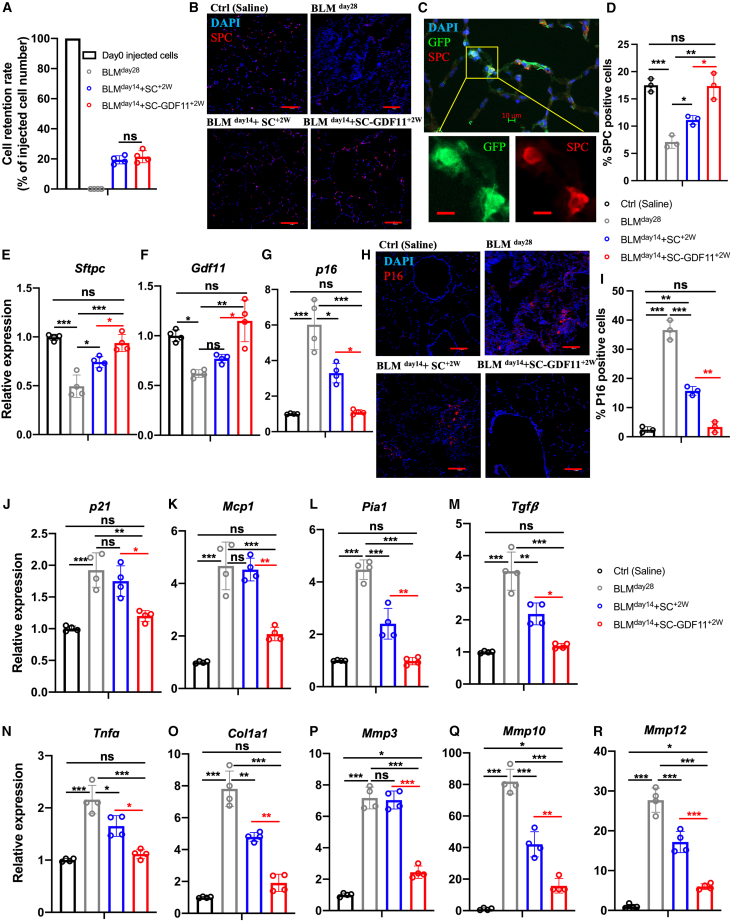
*In situ* restoration of GDF11 levels by cell transplant attenuates age-associated senescence and leads to successful fibrosis resolution (A) Retention rate of the delivered GFP^+^ cells in the recipient lungs (percentage of day 0 injected cells) was calculated using genomic GFP measured at day 28 of BLM treatment (relative to β-actin GFP lungs) measured by PCR. (B) Representative confocal microscopy images of distal airways of all the experimental lungs showing nuclear stain DAPI (blue) and SPC (red). (C) Representative images of the alveolar epithelium of GDF11-GFP cell-treated BLM-injured animals showing nuclear stain DAPI (blue), GFP (green), and SPC (red). (D) Quantification of SPC^+^ cells. (E–G) The expression of *Sftpc* (E), *Gdf11* (F), and *p16* (G) in the lungs of different groups 28 days after saline or BLM treatment, as measured by RT-qPCR comparing fold differences in gene expression in non-injured healthy controls. (H and I) (H) Confocal microscopy images of distal airways of all the experimental lungs showing nuclear stain DAPI (blue) and p16 (red) and (I) quantification of p16^+^ cells. (J–R) Transcript levels of *p21* (J) and proinflammatory and profibrotic SASP factors (K–R), including *Mcp1*, *Pai1*, *Tgfß*, *Tnfα*, *Col1a1*, *Mmp3*, *Mmp10*, and *Mmp12* as measured by RT-qPCR comparing fold differences in the expression in non-injured healthy controls. ∗*p* < 0.05; ∗∗*p* < 0.001; ∗∗∗*p* < 0.0001. Scale bar, 100 μm (B and H); 10 μm (C).

We examined donor cell retention after transplantation. To quantify retention, genomic DNA for GFP was measured using a standard curve. The results showed that the retention rates of both donor cell types were similar, 19.4% ± 2.7% and 21.5% ± 4.0%, respectively (Figure 6A).

After BLM treatment, there were fewer AEC-II cells (as marked by SPC) in comparison to the untreated lungs. However, the lungs that were treated with donor cells showed an elevated number of SPC^+^ cells, with an especially abundant presence in the GDF11-expressing donor cell-treated group (Figures 6B and 6D). The presence of GFP-expressing donor SPC^+^ cells demonstrated their direct contribution to the AEC-II lineage, while no clonal clusters were observed in the lung parenchyma (Figures 6B and 6C). This was further supported by the efficient restoration of the SPC encoding mRNA (*Sftpc*) levels (Figure 6E).

Only the lungs treated with GDF11-expressing cells showed restoration of *Gdf11* mRNA levels, which were comparable to those seen in healthy controls (Figure 6F). This indicates that the GDF11-expressing cells were effective in providing GDF11 *in situ*.

We observed that the elevated transcriptional activation of *p16* induced by BLM was downregulated in the lungs treated with donor cells. This decrease was more pronounced with GDF11-expressing donor cells, reaching levels comparable to those of healthy controls (Figure 6G). Immunostaining also revealed that the number of p16^+^ senescent cells was significantly reduced in these lungs (Figures 6H and 6I).

Similar to p16, the cell-cycle inhibitor p21 is also implicated in cellular senescence.^71^ We observed a significant elevation in *p21* expression in the lungs of BLM-injured mice compared to the control group. However, significant downregulation of *p21* was only evident in the lungs treated with GDF11-expressing cells (Figure 6J). This finding indicates that the restoration of GDF11 activity can also eliminate p21-dependent cell senescence, which may facilitate alveolar regeneration.^71^

Furthermore, we conducted a study on the senescence-associated secretory phenotype (SASP) components in the lungs of all experimental groups. The level of transcripts of proinflammatory and profibrotic SASP factors, including *Mcp1*, *Pai1*, *Tgfß*, *Tnfα*, *Col1a1*, *Mmp3*, *Mmp10*, and *Mmp12*, were measured by RT-qPCR (Figures 6K–6R). Both groups of donor cells showed positive effects. Despite lacking exogenous GDF11, the control donor cells were able to reduce the expression of most of these genes, except for *Mcp1* and *Mmp3* (Figures 6K and 6P). Remarkably, the GDF11-expressing cells showed greater potential in downregulating all these genes, including *Mcp1* and *Mmp3*. Additionally, we observed no signs of tumor formation in mice that received exogenous GDF11 from transplanted GDF11-expressing cells (Figure S4B).

## Discussion

We developed a combination of cell and gene therapy approaches to target human IPF using a mouse model of the disease. For the gene therapy component, we chose GDF11-inducible expression because of the implication of its regenerative potential in certain physiological functions. For the cell therapy component, we generated lung progenitors from engineered ESCs with controlled proliferation by implementing our SC system^49^ into the cells. Upon delivery to injured aging lungs, these progenitor cells can engraft and restore the damaged alveolar epithelium. These cells also serve as a “factory on-site” and can produce the necessary amount of GDF11 to compensate for the natural decline of this factor resulting from aging- or lung-related diseases. This approach effectively mitigates age-associated senescence, which is further exacerbated in disease conditions, resulting in the successful resolution of age-related persistent fibrosis.

Some of the controversy surrounding the function of GDF11 is rooted in its high level of homology with myostatin (GDF8).^22^^,^^23^ Both GDF11 and myostatin are members of the TGF-β superfamily and are involved in tissue regeneration, playing critical roles in regulating cellular processes. They have been extensively studied for their potential therapeutic applications in regenerative medicine. However, their specific regenerative utilities differ significantly. While myostatin primarily regulates muscle growth and restricts muscle stem cell proliferation,^22^^,^^72^ GDF11 demonstrates a broader regenerative capacity beyond the muscle. GDF11 has been shown to promote neurogenesis in the brain, improve cardiovascular function, and facilitate tissue repair across various organs, such as the liver and kidney.^18^^,^^29^ However, myostatin’s effects are focused primarily on skeletal muscle development and maintenance.^22^^,^^73^^,^^74^ Therefore, GDF11’s diverse multi-tissue regenerative properties make it a more appealing candidate for comprehensive therapies, particularly in the context of lung health. Our research indicates a link between decreased levels of GDF11 and impaired fibrosis resolution associated with cellular senescence in aging. Through both *in vitro* and *in vivo* studies, we have demonstrated the regenerative potential of GDF11 in the lung, suggesting broader implications for diseases mediated by age-related senescence,^75^ such as IPF. Therapeutic interventions targeting senescent cells have shown significant promise. The elimination of senescence has been associated with the resolution of fibrosis and improvements in both pulmonary function and overall physical health in animal models.^51^^,^^76^^,^^77^ Our findings demonstrate the anti-senescence effect of GDF11, which aligns with numerous studies across various tissues and models of age-related diseases.^18^^,^^78^^,^^79^ While the underlying mechanism requires further investigation, evidence from previous studies^18^^,^^45^^,^^78^^,^^80^ suggests that the activation of the SMAD2/3, FAK, Akt, and eNOS pathways is likely responsible. Our established GDF11 ESC line could offer broad therapeutic potential by providing diverse cell types for localized delivery of optimal amounts of GDF11. This approach could effectively target senescence across various diseased organs and sites.

The role of GDF11 in fibrosis is a subject of ongoing research and remains somewhat contentious due to its membership in the BMP subfamily of the TGF-β superfamily. The TGF-β subfamily members, such as β1, β2, and β3, are known to be strongly associated with fibrosis by promoting the accumulation of extracellular matrix (ECM) components and contributing to tissue scarring.^81^ While GDF11 is generally thought to have anti-fibrotic effects by promoting BMP signaling,^17^^,^^18^^,^^19^ it is important to note that its role in fibrosis can be complex and context dependent. It can activate both the SMAD1/5/8 pathway, which counterbalances TGF-β signaling in the epithelial-mesenchymal transition and fibrosis, and the profibrotic SMAD2/3 pathway.^13^ Some studies have suggested that GDF11 may also have profibrotic effects in certain conditions or tissues.^16^^,^^20^^,^^21^ The dual roles of GDF11 in fibrosis may be influenced by factors such as the cellular environment, the activity of other signaling pathways, and the specific disease context.

Hence, an appropriate dosage is essential for harnessing the anti-fibrotic potential of GDF11 to specific tissue and disease phenotypes. However, this process could be costly and arduous when using recombinant protein, as demonstrated by numerous studies involving intraperitoneal injection of rGDF11.^46^^,^^47^ High-dose or long-term exposure to rGDF11 can impede therapeutic outcomes and lead to severe adverse effects, such as neurotoxicity, cachexia, and mortality.^24^^,^^47^^,^^48^ These limitations favor using transplanted cells as a localized source of GDF11 production, targeting specific organs or sites of regeneration as needed.

To address the challenges associated with the limited practicality of rGDF11, we have developed a cell-based treatment strategy that allows for localized, controlled, and sustained release of GDF11. This approach involves utilizing transplanted lung progenitor cells that have been differentiated from SC ESCs further engineered to express GDF11 in a controlled manner. We directly transplanted these cells to the diseased site via transtracheal delivery rather than systemic intravenous injection. Animals treated with the GDF11 donor cells showed complete recovery in body weight lost due to BLM-induced injury. Additionally, whole-body PET-CT scans showed no signs of tumor formation (Figure S4B) as expected, since the cells were briefly treated *in vitro* with GCV for 7 days. This treatment activates the kill switch system to eliminate highly proliferative, potentially tumorigenic cells prior to transplantation. Nonetheless, in a clinical setting, patients would require more frequent monitoring and *in vivo* activation of the kill switch system to ensure safety.

In the present study, we chose GDF11 as a prototype regenerative factor. While the existing viewpoint suggests that GDF11 may not be the sole universal facilitator of regeneration or rejuvenation, our methodology provides a flexible approach that can be adapted for various other regenerative and anti-aging agents, such as SIRT,^1^^,^^2^ Klotho,^3^^,^^4^^,^^5^ IGF-1,^6^^,^^7^^,^^8^ PF4.^9^ For all of these agents, our approach has the potential to address practical limitations associated with their use, such as high cost, short half-life, and the need for precise dosing to effectively address specific aging- and disease-related characteristics.

Despite the importance of this proof-of-concept study, certain areas warrant further investigation to enhance therapeutic effectiveness. While senescence was notably reduced by GDF11-expressing cell treatment, the suppression of matrix metalloproteinase (MMP) genes (including Mmp3, Mmp10, and Mmp12) associated with ECM degradation and fibrosis resolution was not fully achieved, and these genes remained elevated compared to healthy controls. Therefore, additional research is necessary to optimize *in vivo* GDF11 expression, including the dosing of transplanted cells, as well as the dosing regimen of dox to optimize the timing/duration of transgene induction to improve therapeutic efficacy.

We demonstrate the potential of GDF11-expressing lung progenitor cells in cell-based therapy for ameliorating age-related IPF in a mouse model. The capacity to deliver GDF11 *in situ* through regulated transgene expression enables a more precise and targeted strategy to address cellular senescence and fibrosis. The effectiveness of this method could have wider implications for treating fibrotic lung conditions linked to aging and senescence.

There is an increasing number of clinical trials exploring cell therapy for the treatment of degenerative diseases. These trials use cells that are derived from either autologous or allogeneic pluripotent stem cells.^82^ Regarding targeting lung diseases, protocols have been developed to generate human lung resident cells with directed *in vitro* differentiation from induced pluripotent stem cells.^83^^,^^84^ However, to advance toward readiness for stem cell-based therapies, two major challenges need to be addressed: ensuring cell therapy safety and promoting acceptance of allogeneic cells without requiring immunosuppression of patients. We have proposed genome-editing solutions for these challenges and developed the SC^49^ and the AlloAccept^85^^,^^86^ systems. The integration of these two systems could lead to the creation of safe and universally applicable off-the-shelf therapeutic products that are accessible to all humans. This advancement could significantly expand the scope of diseases that cell therapy can effectively target, offering an alternative treatment avenue for degenerative diseases on a larger scale within the field of medicine.

## Materials and methods

### Animal husbandry

C57BL/6N (stock no. 005304) mice were purchased from The Jackson Laboratory, and 8- to 10-week-old and 12-month-old mice were used for BLM injury and cell replacement therapy studies. Animals were maintained as an in-house breeding colony under specific pathogen-free conditions. All animal care protocols and procedures were performed in accordance with relevant guidelines and with approval by the Institutional Animal Care and Use Committee of the University Health Network (Toronto, Ontario, Canada).

### BLM administration and cell delivery

For BLM-induced injury, BLM 2.5 U kg^−1^ body weight was administered intratracheally. Donor cells (10^6^ cells in 50 mL PBS) were delivered intratracheally 14 days after injury. Control animals received the same volume of saline without any cells. The mice receiving cells were rotated to ensure equal dispersion of the cell suspension to both lungs.^62^

### Construction of piggyBac vectors

The plasmid containing the cDNA of murine Gdf11 was obtained from the Lunenfeld-Tanenbaum Research Institute Open Freezer repository. To prepare the constructs for expression, the cDNAs were inserted into piggyBac transposon expression vectors using the Gateway cloning kit (Thermo Fisher, catalog no. 12535029) according to the manufacturer’s protocol. In summary, the Gdf11 gene was amplified by extension PCR using PrimeStar HS master mix (Takara, catalog no. R040), with Gateway-compatible attb1/attb2 sites added to the 5′ and 3′ ends, respectively. The resulting product was then recombined into the Gateway pDONr221 vector (Thermo Fisher, catalog no. 12536017), and the transgene insertion was confirmed by sequencing the resulting entry vector using flanking M13 forward and M13 reverse primers. Subsequently, the transgene-containing entry clone was introduced into piggyBac destination vectors via Gateway cloning to produce the final vectors for transgene expression in cells. At each stage of cloning, vectors were introduced into chemically competent DH5alpha cells (Invitrogen), followed by the selection of colonies on LB agar plates supplemented with either kanamycin (Sigma, catalog no. K1377) or ampicillin (Sigma, catalog no. 10835242001). Colonies were then cultured in LB broth (Wisent, catalog no. 809-060-L), and bacterial plasmid DNA was extracted using the Presto Mini Bacterial DNA kit (Geneaid, catalog no. PDH300).

### Fluorescence-activated cell sorting and analysis

For extracellular staining, freshly isolated or cultured cells were suspended and incubated in 0.5% (v/v) fetal bovine serum (FBS)-PBS with an optimally pre-titered mixture of antibodies and relevant isotype controls for approximately 30 min on ice. The labeled cells were then washed and re-suspended at a concentration of 3–5 × 10^6^ cells/mL in 0.5% (v/v) FBS-PBS. Cell viability was assessed by propidium iodide or 4,6-diamidino-2-phenylindole (DAPI; Sigma) staining at a concentration of 1 μg/mL. For intracellular antigen analysis, cells were fixed and stained using the Invitrogen Fix and Perm kit following the manufacturer’s instructions. Sorting was conducted using a MoFlo BRU cell sorter (Becton Dickinson), acquisition was performed using a BD LSRII analyzer (Becton Dickinson), and data were analyzed using FlowJo software.

### Immunofluorescence

Samples were fixed with 4% paraformaldehyde for 30 min and subsequently blocked with a solution containing 5% goat serum and 2% BSA in PBS supplemented with 0.5% Triton X-100 for 1 h. Primary antibodies, diluted in BSA/PBS, were then applied to the samples and left to incubate overnight at 4°C. Following this, secondary antibodies, such as AlexaFluors 488, 532, 546, 633, or 647 (Invitrogen), were applied based on the species for which the primary antibody was raised and incubated for 2 h at room temperature. Nuclear staining was achieved using 2 mg/mL DAPI (Sigma). Stained samples were mounted with immunofluorescent mounting medium (DAKO), and appropriate non-specific immunoglobulin G isotypes were utilized as controls. Immunoreactivities of antigens were visualized as single optical planes using an Olympus Fluoview confocal microscope and analyzed with FV10-ASW 2.0 Viewer software.

### Real-time PCR analysis

Total RNA was extracted from cells or lung tissue utilizing the RNeasy Kit (Qiagen) following the manufacturer’s guidelines. Subsequently, cDNA synthesis and analysis were conducted using Superscript III (Sigma) in accordance with the manufacturer’s instructions. Differential gene expression was assessed using SYBR Green detection (Roche). Real-time PCR reactions were performed in triplicate for each sample. Housekeeping genes were used to standardize gene expression levels. Normalized mRNA levels were presented relative to the corresponding controls.

### Differentiation of distal lung progenitors from engineered ESCs

Engineered ESCs were differentiated into definitive endoderm using activin A (100 ng/mL) and Wnt3a (50 ng/mL; Thermo Fisher) for 3 days after leukemia inhibitory factor withdrawal, following a modified protocol.^87^ Anterior foregut endoderm was induced with SB431542 (10 μM) and rmNoggin (100 ng/mL; R&D Systems). Lung specification was achieved in 3D Matrigel culture using rmWnt3a (100 ng/mL), rmBMP4 (10 ng/mL; R&D Systems), and retinoic acid (100 nM, Sigma). Cells were further differentiated into distal lung progenitors using Small Airway Growth Medium (Lonza) for 2 weeks. To eliminate highly proliferative, potentially tumorigenic cells, cultures were treated with GCV (1 μM, Sigma) from days 7–14. Cells were then purified based on CPM (Invitrogen) surface expression for transplantation.

### PET/CT imaging

#### Mouse preparation and dose administration for imaging

Mice were subjected to overnight fasting while having unrestricted access to water. An intravenous injection of a net dose ranging from 9 to 12 MBq of ^18^F-FDG was administered via a tail vein catheter, followed by a saline flush. Subsequently, mice were anesthetized with 1.5%–2% isoflurane and 1–1.5 L/min oxygen. PET imaging was initiated approximately 1 h post-injection.

#### Imaging

PET/CT imaging was performed on a Mediso NanoScan (Budapest, Hungary) single-photon emission CT/CT/PET system. The CT scan parameters included 50 kVp, 980 μA, and a 300-ms exposure time, followed by a 10-min PET acquisition. Mediso NanoScan Nucline software (version 3.04.012.0000) was used for image acquisition and reconstruction (including automatic PET and CT co-registration). CT scans were reconstructed using the medium voxel and thin-slice thickness settings, resulting in an isotropic voxel size of 78 × 78 × 78 μm. PET images were reconstructed using the standard normal Tera-Tomo 3D reconstruction setting (ordered subset expectation maximization based for 4 subsets, 4 iterations, with attenuation and scatter correction, isotropic voxel size of 400 μm).

#### Image analysis

Image processing and manual and semi-automatic segmentation of the lungs and heart were performed using Siemens Inveon Research Workplace 4.2 (Siemens Medical Solutions).

### Statistical analysis

Statistical analysis was performed using GraphPad Prism 5.0 statistical software. The statistical significance of multiple groups was compared to one another using Tukey’s multiple comparison test ANOVA. A *p* < 0.05 was considered significant.

## Data availability

Relevant data are available directly from the corresponding author.

## Acknowledgments

This study was supported by the Canadian Institutes of Health Research Foundation grant no. 143231 and the Canada Research Chair grant no. 950-230422 to A.N. and the Grosman-Lubin Fellowship Award to L.G. We would like to thank the Spatio-Temporal Targeting and Amplification of Radiation Response (STTARR) program and its affiliated funding agencies, with special thanks to Deborah Scollard, Maria Bisa (from the University Health Network Animal Resources Center), Dr. Luke Kwon, and Teesha Komal for imaging support.

## Author contributions

L.G. designed and performed the experiments, analyzed the data, and wrote the manuscript. P.D. assisted with the *in vivo* animal injury models and cell delivery. E.D.J. assisted with cell culture. E.S. performed the cell injection of the teratoma assay. C.L. monitored the teratoma formation. T.K.W. contributed to the *in vivo* study design and edited the manuscript. A.N. generated the hypothesis, designed the experiments, provided funding, and wrote the manuscript.

## Declaration of interests

A.N. is a shareholder and co-founder of panCELLa Inc.

## Supplemental information

Document S1. Figures S1–S4 and Tables S1 and S2

Document S2. Article plus supplemental information

## References

[1] Yin J., Han P., Song M., Nielsen M., Beach T.G., Serrano G.E., Liang W.S., Caselli R.J., Shi J. (2018). Amyloid-β Increases Tau by Mediating Sirtuin 3 in Alzheimer’s Disease. Mol. Neurobiol..

[2] Wang W., Zheng Y., Sun S., Li W., Song M., Ji Q., Wu Z., Liu Z., Fan Y., Liu F. (2021). A genome-wide CRISPR-based screen identifies KAT7 as a driver of cellular senescence. Sci. Transl. Med..

[3] Kuro-o M., Matsumura Y., Aizawa H., Kawaguchi H., Suga T., Utsugi T., Ohyama Y., Kurabayashi M., Kaname T., Kume E. (1997). Mutation of the mouse klotho gene leads to a syndrome resembling ageing. Nature.

[4] Kurosu H., Yamamoto M., Clark J.D., Pastor J.V., Nandi A., Gurnani P., McGuinness O.P., Chikuda H., Yamaguchi M., Kawaguchi H. (2005). Suppression of Aging in Mice by the Hormone Klotho. Science.

[5] Clemens Z., Sivakumar S., Pius A., Sahu A., Shinde S., Mamiya H., Luketich N., Cui J., Dixit P., Hoeck J.D. (2021). The biphasic and age-dependent impact of klotho on hallmarks of aging and skeletal muscle function. Elife.

[6] Rodríguez-de la Rosa L., Lassaletta L., Calvino M., Murillo-Cuesta S., Varela-Nieto I. (2017). The Role of Insulin-Like Growth Factor 1 in the Progression of Age-Related Hearing Loss. Front. Aging Neurosci..

[7] Mao K., Quipildor G.F., Tabrizian T., Novaj A., Guan F., Walters R.O., Delahaye F., Hubbard G.B., Ikeno Y., Ejima K. (2018). Late-life targeting of the IGF-1 receptor improves healthspan and lifespan in female mice. Nat. Commun..

[8] Vitale G., Pellegrino G., Vollery M., Hofland L.J. (2019). ROLE of IGF-1 System in the Modulation of Longevity: Controversies and New Insights From a Centenarians’ Perspective. Front. Endocrinol..

[9] Leiter O., Brici D., Fletcher S.J., Yong X.L.H., Widagdo J., Matigian N., Schroer A.B., Bieri G., Blackmore D.G., Bartlett P.F. (2023). Platelet-derived exerkine CXCL4/platelet factor 4 rejuvenates hippocampal neurogenesis and restores cognitive function in aged mice. Nat. Commun..

[10] Loffredo F.S., Steinhauser M.L., Jay S.M., Gannon J., Pancoast J.R., Yalamanchi P., Sinha M., Dall’Osso C., Khong D., Shadrach J.L. (2013). Growth differentiation factor 11 is a circulating factor that reverses age-related cardiac hypertrophy. Cell.

[11] Katsimpardi L., Litterman N.K., Schein P.A., Miller C.M., Loffredo F.S., Wojtkiewicz G.R., Chen J.W., Lee R.T., Wagers A.J., Rubin L.L. (2014). Vascular and neurogenic rejuvenation of the aging mouse brain by young systemic factors. Science.

[12] Wang H., Zhang Y., Liu H., Li S. (2023). GDF11, a target of miR-32-5p, suppresses high-glucose-induced mitochondrial dysfunction and apoptosis in HK-2 cells through PI3K/AKT signaling activation. Int. Urol. Nephrol..

[13] Zhang Y., Wei Y., Liu D., Liu F., Li X., Pan L., Pang Y., Chen D. (2017). Role of growth differentiation factor 11 in development, physiology and disease. Oncotarget.

[14] Egerman M.A., Cadena S.M., Gilbert J.A., Meyer A., Nelson H.N., Swalley S.E., Mallozzi C., Jacobi C., Jennings L.L., Clay I. (2015). GDF11 Increases with Age and Inhibits Skeletal Muscle Regeneration. Cell Metab..

[15] Sinha M., Jang Y.C., Oh J., Khong D., Wu E.Y., Manohar R., Miller C., Regalado S.G., Loffredo F.S., Pancoast J.R. (2014). Restoring systemic GDF11 levels reverses age-related dysfunction in mouse skeletal muscle. Science.

[16] Smith S.C., Zhang X., Zhang X., Gross P., Starosta T., Mohsin S., Franti M., Gupta P., Hayes D., Myzithras M. (2015). GDF11 does not rescue aging-related pathological hypertrophy. Circ. Res..

[17] Li X., Ding D., Chen W., Liu Y., Pan H., Hu J. (2021). Growth differentiation factor 11 mitigates cardiac radiotoxicity via activating AMPKα. Free Radic. Res..

[18] Dai Z., Song G., Balakrishnan A., Yang T., Yuan Q., Möbus S., Weiss A.-C., Bentler M., Zhu J., Jiang X. (2020). Growth differentiation factor 11 attenuates liver fibrosis via expansion of liver progenitor cells. Gut.

[19] Frohlich J., Vinciguerra M. (2020). Candidate rejuvenating factor GDF11 and tissue fibrosis: friend or foe?. GeroScience.

[20] Pons M., Koniaris L.G., Moe S.M., Gutierrez J.C., Esquela-Kerscher A., Zimmers T.A. (2018). GDF11 induces kidney fibrosis, renal cell epithelial-to-mesenchymal transition, and kidney dysfunction and failure. Surgery.

[21] Frohlich J., Kovacovicova K., Mazza T., Emma M.R., Cabibi D., Foti M., Sobolewski C., Oben J.A., Peyrou M., Villarroya F. (2020). GDF11 induces mild hepatic fibrosis independent of metabolic health. Aging (Albany NY).

[22] Lian J., Walker R.G., D’Amico A., Vujic A., Mills M.J., Messemer K.A., Mendello K.R., Goldstein J.M., Leacock K.A., Epp S. (2023). Functional substitutions of amino acids that differ between GDF11 and GDF8 impact skeletal development and skeletal muscle. Life Sci. Alliance.

[23] Walker R.G., Czepnik M., Goebel E.J., McCoy J.C., Vujic A., Cho M., Oh J., Aykul S., Walton K.L., Schang G. (2017). Structural basis for potency differences between GDF8 and GDF11. BMC Biol..

[24] Harper S.C., Johnson J., Borghetti G., Zhao H., Wang T., Wallner M., Kubo H., Feldsott E.A., Yang Y., Joo Y. (2018). GDF11 Decreases Pressure Overload–Induced Hypertrophy, but Can Cause Severe Cachexia and Premature Death. Circ. Res..

[25] Ma Y., Liu Y., Han F., Qiu H., Shi J., Huang N., Hou N., Sun X. (2021). Growth differentiation factor 11: a “rejuvenation factor” involved in regulation of age-related diseases?. Aging.

[26] Zhang F., Yang X., Bao Z. (2022). Bioinformatics network analyses of growth differentiation factor 11. Open Life Sci..

[27] Piantadosi P.T., Holmes A. (2023). GDF11 reverses mood and memory declines in aging. Nat. Aging.

[28] Moigneu C., Abdellaoui S., Ramos-Brossier M., Pfaffenseller B., Wollenhaupt-Aguiar B., de Azevedo Cardoso T., Camus C., Chiche A., Kuperwasser N., Azevedo da Silva R. (2023). Systemic GDF11 attenuates depression-like phenotype in aged mice via stimulation of neuronal autophagy. Nat. Aging.

[29] Liu W.-H., Wang X., Wei L., Zou H.-Q. (2023). GDF11 improves ischemia-reperfusion-induced acute kidney injury via regulating macrophage M1/M2 polarization. Kidney Blood Press Res..

[30] López-Ramírez C., Suarez Valdivia L., Rodríguez Portal J. (2018). Causes of pulmonary fibrosis in the elderly. Med. Sci..

[31] Shetty A.K., Kodali M., Upadhya R., Madhu L.N. (2018). Emerging anti-aging strategies - scientific basis and efficacy. Aging Dis..

[32] Murtha L.A., Morten M., Schuliga M.J. (2019). The role of pathological aging in cardiac and pulmonary fibrosis. Aging Dis..

[33] Izbicki G., Segel M.J., Christensen T.G., Conner M.W., Breuer R. (2002). Time course of bleomycin-induced lung fibrosis. Int. J. Exp. Pathol..

[34] Kadam A.H., Schnitzer J.E. (2024). Insights into Disease Progression of Translational Preclinical Rat Model of Interstitial Pulmonary Fibrosis through Endpoint Analysis. Cells.

[35] Gilhodes J.-C., Julé Y., Kreuz S., Stierstorfer B., Stiller D., Wollin L. (2017). Quantification of Pulmonary Fibrosis in a Bleomycin Mouse Model Using Automated Histological Image Analysis. PLoS One.

[36] Mecozzi L., Mambrini M., Ruscitti F., Ferrini E., Ciccimarra R., Ravanetti F., Sverzellati N., Silva M., Ruffini L., Belenkov S. (2020). In-vivo lung fibrosis staging in a bleomycin-mouse model: a new micro-CT guided densitometric approach. Sci. Rep..

[37] Buccardi M., Grandi A., Ferrini E., Buseghin D., Villetti G., Civelli M., Sverzellati N., Aliverti A., Pennati F., Stellari F.F. (2024). Micro-CT-assisted identification of the optimal time-window for antifibrotic treatment in a bleomycin mouse model of long-lasting pulmonary fibrosis. Sci. Rep..

[38] Gems D., Partridge L. (2013). Genetics of Longevity in Model Organisms: Debates and Paradigm Shifts. Annu. Rev. Physiol..

[39] López-Otín C., Blasco M.A., Partridge L., Serrano M., Kroemer G. (2013). The Hallmarks of Aging. Cell.

[40] Vijg J., Campisi J. (2008). Puzzles, promises and a cure for ageing. Nature.

[41] Aoshiba K., Tsuji T., Nagai A. (2003). Bleomycin induces cellular senescence in alveolar epithelial cells. Eur. Respir. J..

[42] Aoshiba K., Tsuji T., Kameyama S., Itoh M., Semba S., Yamaguchi K., Nakamura H. (2013). Senescence-associated secretory phenotype in a mouse model of bleomycin-induced lung injury. Exp. Toxicol. Pathol..

[43] Schafer M.J., White T.A., Iijima K., Haak A.J., Ligresti G., Atkinson E.J., Oberg A.L., Birch J., Salmonowicz H., Zhu Y. (2017). Cellular senescence mediates fibrotic pulmonary disease. Nat. Commun..

[44] Wang D.-X., Zhu X.-D., Ma X.-R., Wang L.-B., Dong Z.-J., Lin R.-R., Cao Y.-N., Zhao J.-W. (2021). Loss of Growth Differentiation Factor 11 Shortens Telomere Length by Downregulating Telomerase Activity. Front. Physiol..

[45] Zhao L., Zhang S., Cui J., Huang W., Wang J., Su F., Chen N., Gong Q. (2019). TERT assists GDF11 to rejuvenate senescent VEGFR2+/CD133+ cells in elderly patients with myocardial infarction. Lab. Invest..

[46] Katsimpardi L., Kuperwasser N., Camus C., Moigneu C., Chiche A., Tolle V., Li H., Kokovay E., Lledo P.M. (2020). Systemic GDF11 stimulates the secretion of adiponectin and induces a calorie restriction-like phenotype in aged mice. Aging Cell.

[47] Jones J.E., Cadena S.M., Gong C., Wang X., Chen Z., Wang S.X., Vickers C., Chen H., Lach-Trifilieff E., Hadcock J.R., Glass D.J. (2018). Supraphysiologic Administration of GDF11 Induces Cachexia in Part by Upregulating GDF15. Cell Rep..

[48] Sutherland B.A., Hadley G., Alexopoulou Z., Lodge T.A., Neuhaus A.A., Couch Y., Kalajian N., Morten K.J., Buchan A.M. (2020). Growth Differentiation Factor-11 Causes Neurotoxicity During Ischemia in vitro. Front. Neurol..

[49] Liang Q., Monetti C., Shutova M.V., Neely E.J., Hacibekiroglu S., Yang H., Kim C., Zhang P., Li C., Nagy K. (2018). Linking a cell-division gene and a suicide gene to define and improve cell therapy safety. Nature.

[50] Moore, B. B., Lawson W.E., Oury T.D., Sisson T.H., Raghavendran K., Hogaboam C.M. (2013). Animal Models of Fibrotic Lung Disease. Am. J. Respir. Cell Mol Biol.

[51] Lehmann M., Korfei M., Mutze K., Klee S., Skronska-Wasek W., Alsafadi H.N., Ota C., Costa R., Schiller H.B., Lindner M. (2017). Senolytic drugs target alveolar epithelial cell function and attenuate experimental lung fibrosis *ex vivo*. Eur. Respir. J..

[52] Redente E.F., Jacobsen K.M., Solomon J.J., Lara A.R., Faubel S., Keith R.C., Henson P.M., Downey G.P., Riches D.W.H. (2011). Age and sex dimorphisms contribute to the severity of bleomycin-induced lung injury and fibrosis. Am. J. Physiol. Lung Cell. Mol. Physiol..

[53] Cho S.J., Stout-Delgado H.W. (2020). Aging and Lung Disease. Annu. Rev. Physiol..

[54] Chow R.D., Majety M., Chen S. (2021). The aging transcriptome and cellular landscape of the human lung in relation to SARS-CoV-2. Nat. Commun..

[55] Koga H., Kaushik S., Cuervo A.M. (2011). Protein homeostasis and aging: The importance of exquisite quality control. Ageing Res. Rev..

[56] Powers E.T., Morimoto R.I., Dillin A., Kelly J.W., Balch W.E. (2009). Biological and Chemical Approaches to Diseases of Proteostasis Deficiency. Annu. Rev. Biochem..

[57] Held N.M., Houtkooper R.H. (2015). Mitochondrial quality control pathways as determinants of metabolic health. Bioessays..

[58] Schumacker P.T., Gillespie M.N., Nakahira K., Choi A.M.K., Crouser E.D., Piantadosi C.A., Bhattacharya J. (2014). Mitochondria in lung biology and pathology: more than just a powerhouse. Am. J. Physiol. Lung Cell. Mol. Physiol..

[59] Wallace D.C. (2013). A mitochondrial bioenergetic etiology of disease. J. Clin. Invest..

[60] Wang Y., Wang G.Z., Rabinovitch P.S., Tabas I. (2014). Macrophage mitochondrial oxidative stress promotes atherosclerosis and nuclear factor-κB–mediated inflammation in macrophages. Circ. Res..

[61] Mei W., Xiang G., Li Y., Li H., Xiang L., Lu J., Xiang L., Dong J., Liu M. (2016). GDF11 Protects against Endothelial Injury and Reduces Atherosclerotic Lesion Formation in Apolipoprotein E-Null Mice. Mol. Ther..

[62] Guo L., Karoubi G., Duchesneau P., Aoki F.G., Shutova M.V., Rogers I., Nagy A., Waddell T.K. (2018). Interrupted reprogramming of alveolar type II cells induces progenitor-like cells that ameliorate pulmonary fibrosis. NPJ Regen. Med..

[63] Naikawadi R.P., Disayabutr S., Mallavia B., Donne M.L., Green G., La J.L., Rock J.R., Looney M.R., Wolters P.J. (2016). Telomere dysfunction in alveolar epithelial cells causes lung remodeling and fibrosis. JCI Insight.

[64] Li Z., Zhang Z., Ren Y., Wang Y., Fang J., Yue H., Ma S., Guan F. (2021). Aging and age-related diseases: from mechanisms to therapeutic strategies. Biogerontology.

[65] Woltjen K., Michael I.P., Mohseni P., Desai R., Mileikovsky M., Hämäläinen R., Cowling R., Wang W., Liu P., Gertsenstein M. (2009). piggyBac transposition reprograms fibroblasts to induced pluripotent stem cells. Nature.

[66] Fujita M., Ye Q., Ouchi H., Harada E., Inoshima I., Kuwano K., Nakanishi Y. (2006). Doxycycline Attenuated Pulmonary Fibrosis Induced by Bleomycin in Mice. Antimicrob. Agents Chemother..

[67] Gotoh S., Ito I., Nagasaki T., Yamamoto Y., Konishi S., Korogi Y., Matsumoto H., Muro S., Hirai T., Funato M. (2014). Generation of Alveolar Epithelial Spheroids via Isolated Progenitor Cells from Human Pluripotent Stem Cells. Stem Cell Rep..

[68] Vande Velde G., Poelmans J., De Langhe E., Hillen A., Vanoirbeek J., Himmelreich U., Lories R.J. (2016). Longitudinal micro-CT provides biomarkers of lung disease and therapy in preclinical models, thereby revealing compensatory changes in lung volume. Dis. Model. Mech..

[69] Tanguy J., Goirand F., Bouchard A., Frenay J., Moreau M., Mothes C., Oudot A., Helbling A., Guillemin M., Bonniaud P. (2021). [18F]FMISO PET/CT imaging of hypoxia as a non-invasive biomarker of disease progression and therapy efficacy in a preclinical model of pulmonary fibrosis: comparison with the [18F]FDG PET/CT approach. Eur. J. Nucl. Med. Mol. Imaging.

[70] Chen R., Zhang K., Chen H., Zhao X., Wang J., Li L., Cong Y., Ju Z., Xu D., Williams B.R.G. (2015). Telomerase Deficiency Causes Alveolar Stem Cell Senescence-associated Low-grade Inflammation in Lungs. J. Biol. Chem..

[71] Lv X., Liu C., Liu S., Li Y., Wang W., Li K., Hua F., Cui B., Zhang X., Yu J. (2022). The cell cycle inhibitor P21 promotes the development of pulmonary fibrosis by suppressing lung alveolar regeneration. Acta Pharm. Sin. B.

[72] Camparini L., Kollipara L., Sinagra G., Loffredo F.S., Sickmann A., Shevchuk O. (2020). Targeted Approach to Distinguish and Determine Absolute Levels of GDF8 and GDF11 in Mouse Serum. Proteomics.

[73] Carnac G., Vernus B., Bonnieu A. (2007). Myostatin in the pathophysiology of skeletal muscle. Curr. Genomics.

[74] Chen M.-M., Zhao Y.-P., Zhao Y., Deng S.-L., Yu K. (2021). Regulation of Myostatin on the Growth and Development of Skeletal Muscle. Front. Cell Dev. Biol..

[75] McHugh D., Gil J. (2018). Senescence and aging: Causes, consequences, and therapeutic avenues. J. Cell Biol..

[76] Hecker L., Logsdon N.J., Kurundkar D., Kurundkar A., Bernard K., Hock T., Meldrum E., Sanders Y.Y., Thannickal V.J. (2014). Reversal of Persistent Fibrosis in Aging by Targeting Nox4-Nrf2 Redox Imbalance. Sci. Transl. Med..

[77] Hashimoto M., Asai A., Kawagishi H., Mikawa R., Iwashita Y., Kanayama K., Sugimoto K., Sato T., Maruyama M., Sugimoto M. (2016). Elimination of p19ARF-expressing cells enhances pulmonary function in mice. JCI Insight.

[78] Finkenzeller G., Stark G.B., Strassburg S. (2015). Growth differentiation factor 11 supports migration and sprouting of endothelial progenitor cells. J. Surg. Res..

[79] Wang D.-X., Dong Z.-J., Deng S.-X., Tian Y.-M., Xiao Y.-J., Li X., Ma X.-R., Li L., Li P., Chang H.-Z. (2023). GDF11 slows excitatory neuronal senescence and brain ageing by repressing p21. Nat. Commun..

[80] Huang H.-T., Liu Z.-C., Wu K.-Q., Gu S.-R., Lu T.-C., Zhong C.-J., Zhou Y.-X. (2019). MiR-92a regulates endothelial progenitor cells (EPCs) by targeting GDF11 via activate SMAD2/3/FAK/Akt/eNOS pathway. Ann. Transl. Med..

[81] Dituri F., Cossu C., Mancarella S., Giannelli G. (2019). The Interactivity between TGFβ and BMP Signaling in Organogenesis, Fibrosis, and Cancer. Cells.

[82] Cheng W., Zeng Y., Wang D. (2022). Stem cell-based therapy for pulmonary fibrosis. Stem Cell Res. Ther..

[83] Sun Y.L., Hennessey E.E., Heins H., Yang P., Villacorta-Martin C., Kwan J., Gopalan K., James M., Emili A., Cole F.S. (2024). Human pluripotent stem cell modeling of alveolar type 2 cell dysfunction caused by ABCA3 mutations. J. Clin. Invest..

[84] Seo H.-R., Han H.-J., Lee Y., Noh Y.-W., Cho S.-J., Kim J.-H. (2022). Human Pluripotent Stem Cell-Derived Alveolar Organoid with Macrophages. Int. J. Mol. Sci..

[85] Harding J., Vintersten-Nagy K., Yang H., Tang J.K., Shutova M., Jong E.D., Lee J.H., Massumi M., Oussenko T., Izadifar Z. (2023). Immune-privileged tissues formed from immunologically cloaked mouse embryonic stem cells survive long term in allogeneic hosts. Nat. Biomed. Eng..

[86] Pavan C., Davidson K.C., Payne N., Frausin S., Hunt C.P.J., Moriarty N., Berrocal Rubio M.Á., Elahi Z., Quattrocchi A.T., Abu-Bonsrah K.D. (2025). A cloaked human stem-cell-derived neural graft capable of functional integration and immune evasion in rodent models. Cell Stem Cell.

[87] Hawkins F., Kramer P., Jacob A., Driver I., Thomas D.C., McCauley K.B., Skvir N., Crane A.M., Kurmann A.A., Hollenberg A.N. (2017). Prospective isolation of NKX2-1-expressing human lung progenitors derived from pluripotent stem cells. J. Clin. Invest..

