# Supplementary material for: GDF11-secreting cell transplant efficiently ameliorates age-related pulmonary fibrosis
Source: Mol Ther. 2025 Jul 16;33(10):5131–48. doi: 10.1016/j.ymthe.2025.07.003 (PMC12848227; doi:10.1016/j.ymthe.2025.07.003)
Supplement: Document S1. Figures S1–S4 and Tables S1 and S2 [file mmc1.pdf]

YMTHE, Volume *33*

Supplemental Information

GDF11-secreting cell transplant efficiently ameliorates age-related pulmonary fibrosis

Li Guo, Pascal Duchesneau, Eric D. Jong, Evan Sawula, Chengjin Li, Thomas K. Waddell, and Andras Nagy

Supplemental figures and tables

Figure S1

a

Gene symbol	Gene name	Cellular function
Gapdh	glyceraldehyde-3- phosphate dehydrogenase	Catalyzes the reversible oxidative phosphorylation of glyceraldehyde-3-phosphate in glycolysis
B2m	β-2-microglobulin	Beta-chain of major histocompatibility complex class I molecules. Involved in immune response.
Eef2	Eukaryotic Translation Elongation Factor 2	Protein Synthesis
Hprt	Hypoxanthine phosphoribosyltransferase 1	Purine synthesis through the purine salvage pathway
Rpl13a	Ribosomal protein L13a	Structural component of the large 60S ribosomal subunit
Ppia	Peptidylprolyl isomerase A	Protein coding, a cyclosporin binding-protein

b

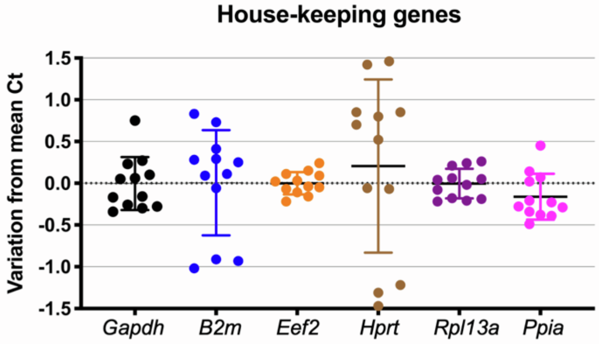

c

	Summary	P Value
**Gapdh**		
Young (Saline) vs. Young (Bleomycin)	ns	0.2606
Young (Saline) vs. Old (Saline)	ns	0.2195
Young (Saline) vs. Old (Bleomycin)	ns	0.1833
Young (Bleomycin) vs. Old (Saline)	ns	0.9997
Young (Bleomycin) vs. Old (Bleomycin)	**	0.0016
Old (Saline) vs. Old (Bleomycin)	**	0.0012
**B2m**		
Young (Saline) vs. Young (Bleomycin)	****	<0.0001
Young (Saline) vs. Old (Saline)	ns	0.9685
Young (Saline) vs. Old (Bleomycin)	ns	0.1295
Young (Bleomycin) vs. Old (Saline)	****	<0.0001
Young (Bleomycin) vs. Old (Bleomycin)	****	<0.0001
Old (Saline) vs. Old (Bleomycin)	ns	0.2968
**Eef2**		
Young (Saline) vs. Young (Bleomycin)	*ns*	*0.5409*
Young (Saline) vs. Old (Saline)	ns	0.2694
Young (Saline) vs. Old (Bleomycin)	ns	0.9213
Young (Bleomycin) vs. Old (Saline)	ns	0.9601
Young (Bleomycin) vs. Old (Bleomycin)	ns	0.8923
Old (Saline) vs. Old (Bleomycin)	ns	0.6271

**Hprt**		
Young (Saline) vs. Young (Bleomycin)	****	<0.0001
Young (Saline) vs. Old (Saline)	*	0.0102
Young (Saline) vs. Old (Bleomycin)	*	0.039
Young (Bleomycin) vs. Old (Saline)	****	<0.0001
Young (Bleomycin) vs. Old (Bleomycin)	****	<0.0001
Old (Saline) vs. Old (Bleomycin)	****	<0.0001
**Rpl13a**		
Young (Saline) vs. Young (Bleomycin)	ns	0.7793
Young (Saline) vs. Old (Saline)	ns	0.9994
Young (Saline) vs. Old (Bleomycin)	ns	0.1458
Young (Bleomycin) vs. Old (Saline)	ns	0.7115
Young (Bleomycin) vs. Old (Bleomycin)	ns	0.6148
Old (Saline) vs. Old (Bleomycin)	ns	0.1146
**Ppia**		
Young (Saline) vs. Young (Bleomycin)	ns	0.8497
Young (Saline) vs. Old (Saline)	ns	0.4109
Young (Saline) vs. Old (Bleomycin)	ns	0.6997
Young (Bleomycin) vs. Old (Saline)	ns	0.8761
Young (Bleomycin) vs. Old (Bleomycin)	ns	0.2436
Old (Saline) vs. Old (Bleomycin)	ns	0.0519

Figure S1. Selection of house-keeping genes that are stably expressed in the lungs during aging in both physiological and pathological conditions **a** The list of candidate house-keeping genes; **b** The plots represent variation from the mean RT-qPCR threshold (Ct) values of candidate housekeeping genes starting from equal amounts of RNA obtained from lung tissues of young (8-

10-week-old) and old mice (12-month-old) at day 28 post saline and BLM-administration, as measured by RT-qPCR; **c** The summary of statistical analysis.

Figure S2

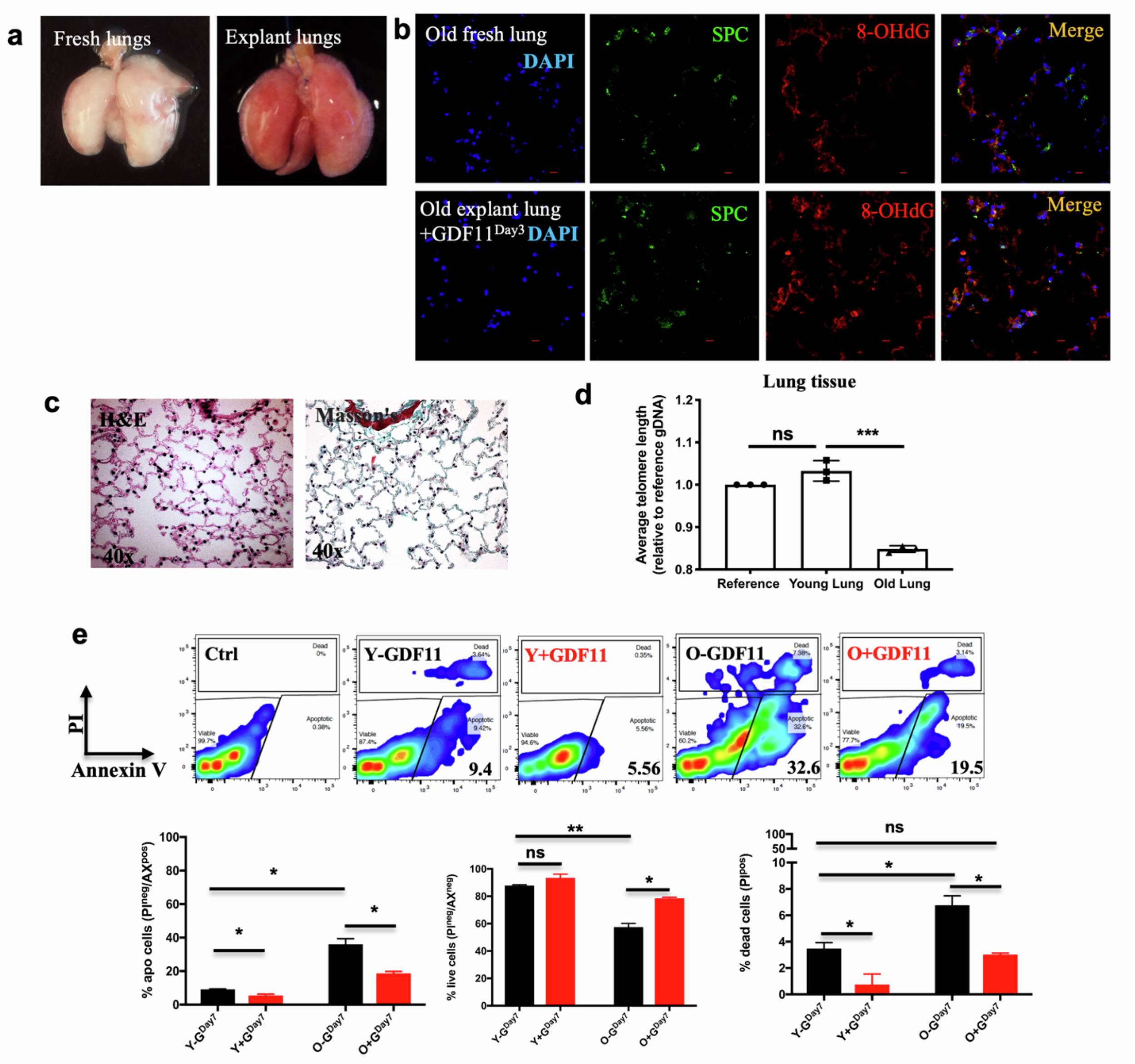

Figure S2. Exogenous GDF11 can partially ameliorate age-related cellular deterioration in the distal lung **a** Representative images of fresh and explant lungs of old mice (12-month-old); **b** Representative confocal microscopy images of distal airways of explant lungs of old mice treated with or without GDF11 recombinant protein for 3 days showing nuclear stain DAPI (blue), SPC (green) and 8-OHdG (red); **c** Representative images of Hematoxylin and Eosin and Masson's trichrome staining of the explant lungs treated with GDF11 recombinant protein for 7 days; **d** Expression levels of average telomere length in lung tissues obtained from young (8-10-week-old) and old (12-month-old) mice each, as measured by RT-qPCR comparing fold differences in the

expression in reference tissues of 8-10 weeks mice; **e** Flow cytometric analysis of PI and Annexin V expression in cells cultured in each condition, and quantification of live, dead and apoptotic cells. *p < 0.05; **p < 0.001; ***p < 0.0001. In **d**, the data are representative of a minimum of three independent biological replicates. Scale bar, 10 µm (**b**).

Figure S3

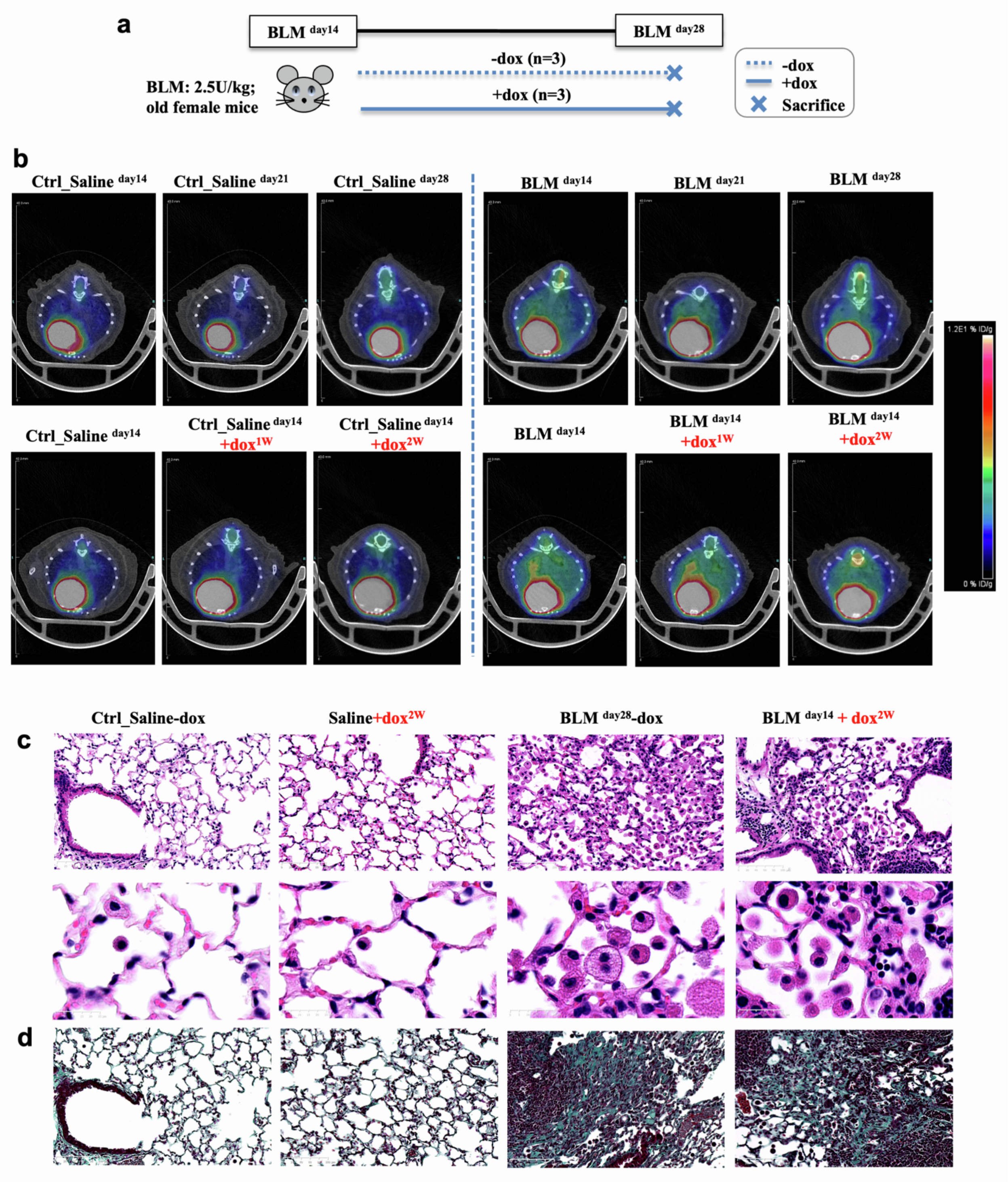

Figure S3. Established fibrosis in aged mice was not affected by the doxycycline diet employed for in-*vivo* transgene activation **a** The experimental scheme of the *in-vivo* study to evaluate the effect of doxycycline diet on fibrosis resolution in the mouse IPF model; **b** Representative PET-CT scan images showing ^{18}F-FDG uptake in the lungs of different groups; Hematoxylin–eosin **c** and Masson's trichrome **d** stained of lung sections showed inflammatory cell infiltration and collagen deposition in BLM-induced fibrotic lungs, regardless of regular or doxycycline diet.

Figure S4

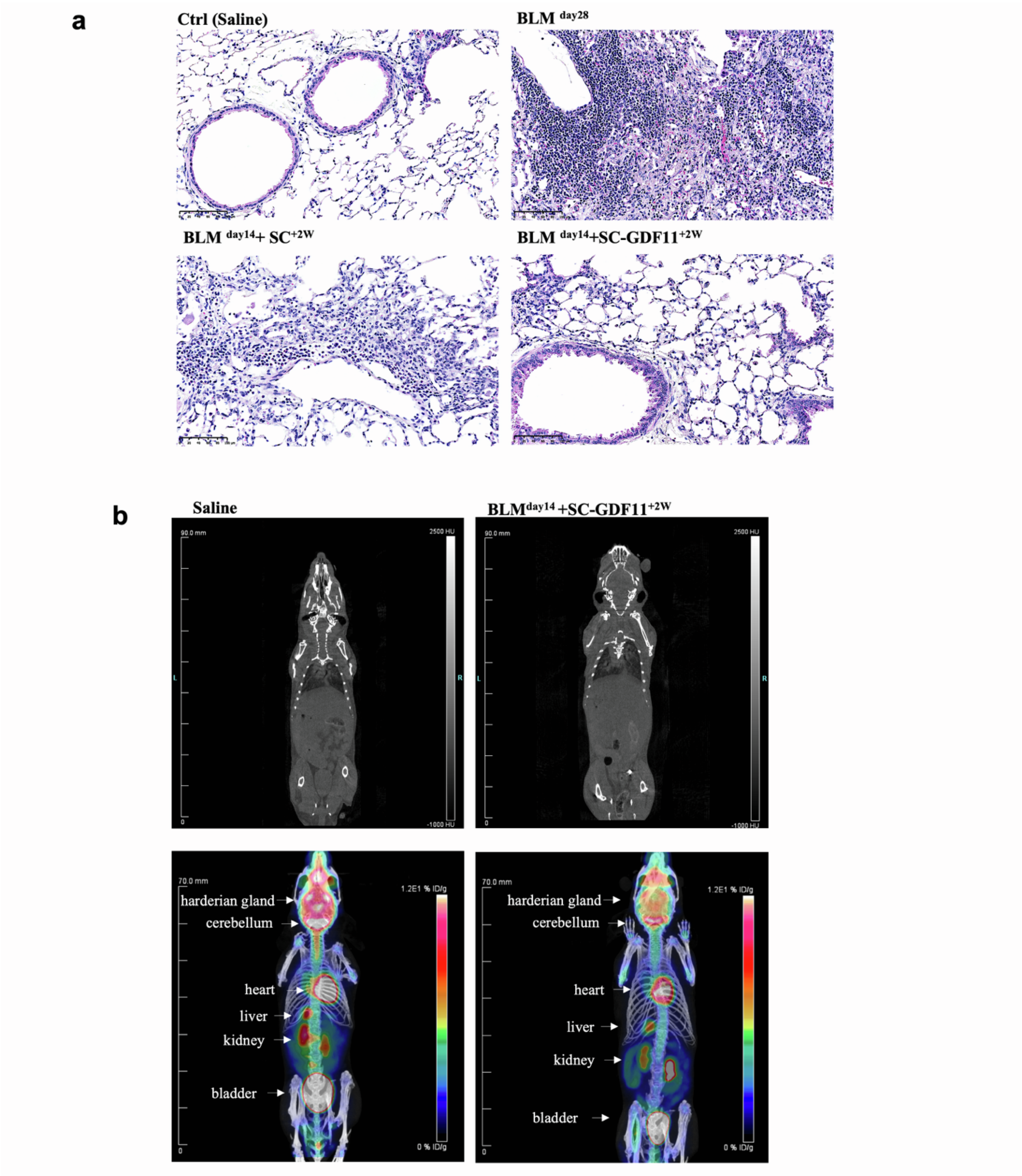

Figure S4. No signs of tumour formation in mice that received exogenous GDF11 from transplanted GDF11-expressing cells **a** Representative images of Hematoxylin–eosin staining from all the experimental groups after 28 days of BLM-induced pulmonary fibrosis; **b** Representative whole-body micro-CT (top panel) and PET-CT (bottom panel) scan images of non-

injured healthy control mice and GDF11 cell-treated injured recipients (BLM^{day14} +$SC\text{-}GDF11^{+2W}$) showing an expected biodistribution of radiotracer with activity in normal tissue and physiologic radiotracer excretion. Scale bar, 100 µm (**a**).

Table S1. List of antibodies used in the studies

Antibody name	Company	Cat. #	Validation
c-KIT	Thermofisher	47-1172-82	ES and ES-derived cells
CXCR4	Biolegend	146508	ES and ES-derived cells
GDF11	Cedarlane	orb1183719	Adult lung tissue;
GDF11	Cedarlane	orb101175	ES and ES-derived cells
8-OHdG	Abcam	ab62623	Adult lung tissue
**γ**H2AX	Abcam	ab11174	Adult lung tissue and cells
CD45	BD Pharmingen	553081	Adult lung cells
CD31	BD Pharmingen	553373	Adult lung cells
EpCAM	Abcam	ab95641	Adult lung cells; ES-derived cells
Nanog	Thermofisher	50-5761-82	ES cells
	Abcam	ab80892	ES cells
OCT4	Abcam	ab184665	ES cells
NKX2.1	Abcam	ab242428	ES-derived cells
SPC	Millipore	AB3786	Adult lung tissue
GFP	Thermo Fisher	A-21311	Adult lung tissue
P16	Abcam	ab54210	Adult lung tissue and cells
a-SMA	Abcam	ab124964	Adult lung tissue

Table S2. Tabulated list of all RT-qPCR primers used in the studies

Gene name	Primer sequence 5'-3'		Length
Gdf11	Forward	CCGGCGTCACATCCGTATC	19
	Reverse	ACTTGCTTGAAGTCGATGCTC	21
S100a4	Forward	TCCACAAATACTCAGGCAAAGAG	23
	Reverse	GCAGCTCCCTGGTCAGTAG	19
p16	Forward	CTCTGCTCTTGGGATTGGC	19
	Reverse	GTGCGATATTTGCGTTCCG	19
Gapdh	Forward	AGGTCGGTGTGAACGGATTTG	20
	Reverse	TGTAGACCATGTAGTTGAGGTCA	21
B2m	Forward	TGACCGGCTTGTATGCTATC	20
	Reverse	CAGTGTGAGCCAGGATATAG	20
Eef2	Forward	TGTCAGTCATCGCCCATGTG	19
	Reverse	CATCCTTGCGAGTGTCAGTGA	20
Hprt	Forward	AGCAGGTCAGCAAAGAACT	19
	Reverse	CCTCATGGACTGATTATGGACA	22
Rpl13a	Forward	CTCAAGGTCGTGCGTCTGAA	20
	Reverse	TGGCTGTCACTGCCTGGTACT	21
Ppia	Forward	GGGTTCCTCCTTTCACAGAA	20
	Reverse	GATGCCAGGACCTGTATGCT	20
p21	Forward	CGGTGTCAGAGTCTAGGGGA	18
	Reverse	ATCACCAGGATTGGACATGG	24
Gadd45b	Forward	CGGCCAAACTGATGAATGT	21
	Reverse	TCTGCAGAGCGATATCATCC	23
Atf3	Forward	CTCTGGCCGTTCTCTGGA	24
	Reverse	GGTCGCACTGACTTCTGAGG	22
Il6	Forward	TCCTTAGCCACTCCTTCTGT	20
	Reverse	AGCCAGAGTCCTTCAGAGA	19
Mmp13	Forward	GGACTCACTGTTGGTCCCTG	20
	Reverse	GGATTCCCGCAAGAGTCACA	20
Sftpc	Forward	GCAAAGAGGTCCTGATGGAG	20
	Reverse	GCAGTAGGTTCCTGGAGCTG	20
Mcp1	Forward	AACTACAGCTTCTTTGGGACA	21
	Reverse	CATCCACGTGTTGGCTCA	18
Pia1	Forward	CGTGTCAGCTCGTCTACAG	19
	Reverse	CTATGGTGAAACAGGTGGACT	21
*Tgf**β***	Forward	CCGAATGTCTGACGTATTGAAGA	23
	Reverse	GCGGACTACTATGCTAAAGAGG	22

Tnfa	Forward	TCTTTGAGATCCATGCCGTTG	21
	Reverse	AGACCCTCACACTCAGATCA	20
Col1a1	Forward	CATTGTGTATGCAGCTGACTTC	22
	Reverse	CGCAAAGAGTCTACATGTCTAGG	23
Mmp3	Forward	TGTGGAGGACTTGTAGACTGG	21
	Reverse	GATGAACGATGGACAGAGGATG	22
Mmp10	Forward	TGTTGCTCTTCAGTATGTGTGT	22
	Reverse	CCAGGAATTGAGCCACAAGT	20
Mmp12	Forward	GCTCCTGCCTCACATCATAC	20
	Reverse	GGCTTCTCTGCATCTGTGAA	20
